# Experimentally-validated correlation analysis reveals new anaerobic methane oxidation partnerships with consortium-level heterogeneity in diazotrophy

**DOI:** 10.1038/s41396-020-00757-1

**Published:** 2020-10-15

**Authors:** Kyle S. Metcalfe, Ranjani Murali, Sean W. Mullin, Stephanie A. Connon, Victoria J. Orphan

**Affiliations:** Division of Geological and Planetary Sciences, California Institute of Technology, 1200 E California Blvd, Mail Code 170-25, Pasadena CA, 91125 USA

**Keywords:** Stable isotope analysis, Symbiosis, Metagenomics, Microbial ecology, Water microbiology

## Abstract

Archaeal anaerobic methanotrophs (“ANME”) and sulfate-reducing Deltaproteobacteria (“SRB”) form symbiotic multicellular consortia capable of anaerobic methane oxidation (AOM), and in so doing modulate methane flux from marine sediments. The specificity with which ANME associate with particular SRB partners in situ, however, is poorly understood. To characterize partnership specificity in ANME-SRB consortia, we applied the correlation inference technique SparCC to 310 16S rRNA amplicon libraries prepared from Costa Rica seep sediment samples, uncovering a strong positive correlation between ANME-2b and members of a clade of Deltaproteobacteria we termed SEEP-SRB1g. We confirmed this association by examining 16S rRNA diversity in individual ANME-SRB consortia sorted using flow cytometry and by imaging ANME-SRB consortia with fluorescence in situ hybridization (FISH) microscopy using newly-designed probes targeting the SEEP-SRB1g clade. Analysis of genome bins belonging to SEEP-SRB1g revealed the presence of a complete *nifHDK* operon required for diazotrophy, unusual in published genomes of ANME-associated SRB. Active expression of *nifH* in SEEP-SRB1g within ANME-2b—SEEP-SRB1g consortia was then demonstrated by microscopy using hybridization chain reaction (HCR-) FISH targeting *nifH* transcripts and diazotrophic activity was documented by FISH-nanoSIMS experiments. NanoSIMS analysis of ANME-2b—SEEP-SRB1g consortia incubated with a headspace containing CH_4_ and ^15^N_2_ revealed differences in cellular ^15^N-enrichment between the two partners that varied between individual consortia, with SEEP-SRB1g cells enriched in ^15^N relative to ANME-2b in one consortium and the opposite pattern observed in others, indicating both ANME-2b and SEEP-SRB1g are capable of nitrogen fixation, but with consortium-specific variation in whether the archaea or bacterial partner is the dominant diazotroph.

## Introduction

The partnership between anaerobic, methanotrophic Archaea (ANME) and their associated sulfate-reducing bacteria (SRB) is one of the most biogeochemically-important symbioses in the deep-sea methane cycle [1, 2]. As a critical component of methane seep ecosystems, multicellular consortia of ANME and associated SRB consume a significant fraction of the methane produced in marine sediments, using sulfate as a terminal electron acceptor to perform the anaerobic oxidation of methane (AOM) [1–4]. ANME-SRB consortia are thought to perform AOM through the direct extracellular transfer of electrons between ANME and SRB [5–7]. Along with symbiotic extracellular electron transfer, ANME-SRB consortia also exhibit other traits of mutualism such as the sharing of nutrients. For example, members of the ANME-2 clade have been reported to fix and share N with partner bacteria [8–11], but the extent to which diazotrophic capability might vary across the diverse clades of ANME and associated SRB is the focus of ongoing research.

Comparative studies of ANME [12] and associated SRB [13, 14] genomes from multiple ANME-SRB consortia have revealed significant diversity across clades, particularly for SRB genomes falling within subclades of the Desulfobacteraceae SEEP-SRB1a [14], common SRB partners to ANME [15]. However, the implications of symbiont diversity for metabolic adaptation in ANME-SRB consortia are obscured by the absence of clearly-established ANME-SRB pairings in the environment. A framework defining these pairings would address this gap in knowledge. Establishing this framework for partnership specificity in ANME-SRB consortia—being the preference that certain ANME exhibit for specific SRB partners—would shed light on the extent to which ANME or SRB physiology may differ in consortia constituted of different ANME-SRB pairs.

As an aspect of ANME or SRB physiology that may differ in different ANME-SRB pairings, nitrogen anabolism has been observed to be involved in the symbiotic relationship between partners [8, 9] and has been shown to influence niche differentiation of different ANME-SRB consortia via nitrate assimilation ability [16]. Previous evidence documenting active diazotrophy by AOM consortia from cDNA libraries of *nifH* [8] and ^15^N_2_ stable isotope probing with FISH-nanoSIMS, indicated that the methanotrophic ANME-2 archaea fixed more nitrogen than SRB in consortia and may supply fixed nitrogen to their syntrophic partners [8–10]. The diazotrophic potential of syntrophic SRB, however, and their role in nitrogen fixation within consortia is poorly understood. Evidence from SRB genomes [14] and the expression of unidentified nitrogenase sequences in methane seep sediments [8] suggested that some seep-associated SRB may also fix nitrogen, opening up the possibility of variation in diazotrophic activity among taxonomically-distinct ANME-SRB consortia.

Previous research characterizing the diversity of partnerships in ANME-SRB consortia have employed fluorescence microscopy, magnetic separation by magneto-FISH, and single-cell sorting techniques (e.g., BONCAT-FACS) that are robust against false positives, but are often limited in statistical power. Fluorescence in situ hybridization (FISH) has helped to establish the diversity of ANME-bacterial associations, with ANME constituting four diverse polyphyletic clades within the Methanomicrobia: ANME-1a/b [4, 17–20], ANME-2a,b,c [3, 20–22], ANME-2d [23, 24], and ANME-3 [20, 25, 26]. ANME-associated SRB have also observed by FISH to be diverse, representing several clades of Deltaproteobacteria including the *Desulfococcus/Desulfosarcina* (DSS) clade [3–6, 15, 19–22, 27–33], two separate subclades within the Desulfobulbaceae [16, 25, 26], a deeply-branching group termed the SEEP-SRB2 [34], and a thermophilic clade of Desulfobacteraceae known as HotSeep-1 [34, 35]. These FISH studies documented associations for different ANME-SRB consortia, including partnerships between members of ANME-1 and SEEP-SRB2 [13] or HotSeep-1 [7, 13, 35], ANME-2a and SEEP-SRB1a [15], ANME-2c and SEEP-SRB1a [5], SEEP-SRB2 [13, 34], or Desulfobulbaceae [29], and ANME-3 and SEEP-SRB1a [15] or Desulfobulbaceae [25, 26]. Conspicuously, SRB found in consortia with ANME-2b have only been identified broadly as members of the Deltaproteobacteria targeted by the probe S-C-dProt-0495-a-A-18 (often referred to as Δ495) [5, 31, 36], leaving little known about the specific identity of this SRB partner. Visualizing ANME-SRB partnerships by FISH has been a valuable aspect of AOM research, but FISH requires the design of probes with sufficient specificity to identify partner organisms and thus will only detect partnerships consisting of taxa for which phylogenetic information is known [22]. Magneto-FISH [29, 37, 38] or BONCAT-enabled fluorescence-activated cell sorting (BONCAT-FACS) of single ANME-SRB consortia [39] complement FISH experiments by physical capture (via magnetic beads or flow cytometry, respectively) and sequencing of ANME and associated SRB from sediment samples. These studies corroborated some of the patterns observed from FISH experiments, showing associations between ANME-2 and diverse members of the DSS [39]. Magneto-FISH and BONCAT-FACS observations of ANME-SRB pairings are also highly robust against false positives but can lack the statistical power conferred by more high-throughput approaches that is necessary to establish a general framework for partnership specificity.

Recently, a number of correlation analysis techniques have been introduced in molecular microbial ecology studies, providing information about patterns of co-occurrence between 16S rRNA operational taxonomic units (OTUs) or amplicon sequence variants (ASVs) recovered from environmental diversity surveys [40–43]. Correlation analysis performed on 16S rRNA amplicon surveys provides a complementary method to Magneto-FISH and/or BONCAT-FACS that can be used to develop hypotheses about potential microbial interactions. While predictions of co-occurrence between phylotypes from these correlation analysis techniques have been reported in a number of diverse environments, they are rarely validated through independent approaches, with a few notable exceptions (e.g., [44]).

Here, we present a framework for ANME-SRB partnership specificity, using correlation analysis of 16S rRNA amplicon sequences from a large-scale survey of seafloor methane seep sediments near Costa Rica to predict potential ANME-SRB partnerships. A partnership between ANME-2b and members of an SRB group previously not known to associate with ANME (SEEP-SRB1g) was hypothesized by correlation analysis and independently assessed by FISH and by analysis of amplicon data from Hatzenpichler et al. [39] of BONCAT-FACS-sorted ANME-SRB consortia. With this new framework, we were able to identify a novel partnership between ANME-2b and SEEP-SRB1g and map predicted physiological traits of SEEP-SRB1g genomes onto partnership specificity with ANME-2b. Our approach led us to formulate new hypotheses regarding how SEEP-SRB1g physiology may complement ANME-2b physiology, focusing on nitrogen fixation in SEEP-SRB1g. We demonstrate in this study that the symbiotic relationship between ANME and associated SRB can vary depending on the nature of the partner taxa and affirm the importance of characterizing individual symbiont pairings in understanding AOM symbiosis.

## Materials and methods

Here, we present an abridged description of the methods used in this study. A full description can be found in the Supplemental Materials and Methods.

### Sample origin and processing

Pushcore samples of seafloor sediment were collected by DSV *Alvin* during the May 20–June 11, 2017 ROC HITS Expedition (AT37-13) aboard R/V *Atlantis* to methane seep sites southwest of Costa Rica [45–47]. After retrieval from the seafloor, sediment pushcores were extruded aboard R/V *Atlantis* and sectioned at 1–3 cm intervals for geochemistry and microbiological sampling using published protocols [21, 48]. Samples for DNA extraction were immediately frozen in liquid N_2_ and stored at −80 °C. Samples for microscopy were fixed in 2% paraformaldehyde for 24 h at 4 °C. A full list of samples used in this study can be found in Supplementary Table 1 and additional location and geochemical data can be found at https://www.bco-dmo.org/dataset/715706.

### DNA extraction and Illumina 16S rRNA amplicon sequencing

DNA was extracted from 310 samples of Costa Rican methane seep sediments and seep carbonates (Supplementary Table 1) using the Qiagen PowerSoil DNA Isolation Kit 12888 following manufacturer directions modified for sediment and carbonate samples [21, 49]. The V4-V5 region of the 16S rRNA gene was amplified using archaeal/bacterial primers, 515 F (5′-GTGYCAGCMGCCGCGGTAA-3′) and 926 R (5′-CCGYCAATTYMTTTRAGTTT-3′) with Illumina adapters [50]. PCR reaction mix was set up in duplicate for each sample with New England Biolabs Q5 Hot Start High-Fidelity 2x Master Mix in a 15 µL reaction volume with annealing conditions of 54 °C for 30 cycles. Duplicate PCR samples were then pooled and 2.5 µL of each product was barcoded with Illumina NexteraXT index 2 Primers that include unique 8-bp barcodes. Amplification with barcoded primers used annealing conditions of 66 °C and 10 cycles. Barcoded samples were combined into a single tube and purified with Qiagen PCR Purification Kit 28104 before submission to Laragen (Culver City, CA, USA) for 2 × 250 bp paired-end analysis on Illumina’s MiSeq platform. Sequence data were submitted to the NCBI Sequence Read Archive as Bioproject PRJNA623020. Sequence data were processed in QIIME version 1.8.0 [51] following Mason et al. [52]. Sequences were clustered into de novo operational taxonomic units (OTUs) with 99% similarity [53], and taxonomy was assigned using the SILVA 119 database [54], which uses NCBI rather than GTDB taxonomy. Known contaminants in PCR reagents as determined by analysis of negative controls run with each MiSeq set were also removed (see Supplementary Materials and Methods) along with rare OTUs not present in any given library at a level of at least 10 reads. The produced table of OTUs detected in the 310 methane seep sediment and seep carbonate amplicon libraries was analyzed using the correlation algorithm SparCC [41].

To examine phylogenetic placement of SRB 16S rRNA gene amplicon sequences predicted by network analysis to associate with particular ANME subgroup amplicon sequences, a phylogeny was constructed using RAxML-HPC [55] on XSEDE [56] using the CIPRES Science Gateway [57] from full-length 16S rRNA sequences of Deltaproteobacteria aligned by MUSCLE [58]. Genomes downloaded from the IMG/M database were searched using tblastn. Chlorophyllide reductase BchX (WP011566468) was used as a query sequence for a tblastn *nifH* search using BLAST+. BchX was used as the query sequence to recover divergent *nifH* sequences covering the diversity of all *nifH* clades, following the approach of Dekas et al. [8]. Genome trees were constructed using the Anvi’o platform [59] using HMM profiles from a subset [60] of ribosomal protein sequences and visualized in iTOL [61].

### FISH probe design and microscopy

A new FISH probe was designed in ARB [62]. This probe, hereafter referred to as Seep1g-1443 (Supplementary Table 2), was designed to complement and target 16S rRNA sequences in a monophyletic “*Desulfococcus* sp.” clade. Based on phylogenetic analysis (see below), this clade was renamed SEEP-SRB1g, following the naming scheme of Schreiber et al. [15]. Seep1g-1443 was ordered from Integrated DNA Technologies (Coralville, IA, USA). FISH reaction conditions were optimized for Seep1g-1443, with optimal formamide stringency found to be 35% (Supplementary Fig. 1). FISH and hybridization chain reaction (HCR-) FISH was performed on fixed ANME-SRB consortia using previously published density separation and FISH protocols [22], using a selection of following FISH probes: Seep1g (Alexa488; this work), Seep1a-1441 (cy5; [15]), ANME-2a-828 (cy3(3′); M. Aoki, personal communication), ANME-2b-729 (cy3; [39]), and ANME-2c-760 (cy3; [20]). FISH was performed overnight (18 h) using modifications (G. Chadwick, personal communication) to previously-published protocols [29, 39, 63, 64]. Structured-illumination microscopy (SIM) was performed on FISH and HCR-FISH (see below) experiments to image ANME-SRB consortia using the Elyra PS.1 SIM platform (Zeiss, Germany) and an alpha Plan-APOCHROMAT 100X/1.46 Oil DIC M27 objective. Zen Black software (Zeiss) was used to construct final images from the structured-illumination data.

### mRNA imaging using HCR-FISH

Hybridization chain reaction FISH (HCR-FISH) is a powerful technique to amplify signal from FISH probes [65, 66]. The protocol used here was modified from Yamaguchi and coworkers [67]. *nifH* initiators, purchased from Molecular Technologies (Pasadena, CA, USA; probe identifier “nifH 3793/D933”) or designed in-house (Supplementary Table 2) and ordered from Integrated DNA Technologies, were hybridized to fixed ANME-SRB consortia. Hairpins B1H1 and B1H2 with attached Alexa647 fluorophores (Molecular Technologies) were added separately to two 45 µL volumes of amplification buffer in PCR tubes and snap cooled by placement in a C1000 Touch Thermal Cycler (BioRad, Hercules, CA, USA) for 3 min at 95 °C. After 30 min at room temperature, hairpins were mixed and placed in PCR tubes along with hybridized ANME-SRB consortia. Amplification was performed for 15 min at 35 °C. Similar results were observed when the HCR-FISH v3.0 protocol established by Choi et al. [68] was used. ANME-SRB consortia subjected to HCR-FISH experiments were imaged using the Elyra PS.1 SIM platform (Zeiss, Germany) as mentioned above. In all cases, the FITC channel was subject to a 500 ms exposure time, TRITC to 200 ms, and cy5 to 1000 ms. Colocalization of signal was analyzed in ImageJ using the Colocalization Finder and JaCoP plugin [69]. These plugins were used to compute the Pearson’s cross-correlation coefficient (PC) and Manders’ colocalization coefficients (M1, M2). In addition, pairwise correlations between channels were visualized using scatterplots of pixel intensity.

### Stable isotope probing and nanoSIMS

Methane seep sediments containing abundant ANME-2b and SEEP-SRB1g consortia (Supplementary Fig. 2) were used in stable isotope probing (SIP) experiments to test for diazotrophic activity by SEEP-SRB1g. SIP incubations (Supplementary Table 3) were prepared by sparging source bottles and 30 mL serum bottles with N_2_ and mixing 5 mL of sediment with 5 mL N_2_-sparged artificial seawater without a N source. N sources were removed from the sediment slurry by washing with artificial seawater without an N source (see Supplementary Materials and Methods). Two anoxic incubations were pressurized with 2.8 bar CH_4_ with 1.2 mL ^15^N_2_ (Cambridge Isotopes, Tewksbury, MA, part # NLM-363-PK, lot # l-21065) at 1 bar, approximately equivalent to 2% headspace in 20 mL CH_4_ at 2.8 bar (Supplementary Table 3). Potential ^15^NH_4_^+^ contamination in ^15^N_2_ stocks have been previously reported and can lead to spurious results in nitrogen fixation experiments. We did not test for fixed N in the specific reagent bottle used in these experiments. However, previous comparisons of ^15^N_2_ stocks identify those from Cambridge Isotopes as among the least-contaminated ^15^N_2_ stocks available [70]. Positive control incubations (*n* = 2) were amended with 500 µM ^15^NH_4_Cl and were pressurized with 2.8 bar CH_4_ and 1.2 mL natural-abundance N_2_ at 1 bar. Incubations were periodically checked for AOM activity via sulfide production using the Cline assay [71] and were chemically fixed for FISH-nanoSIMS analysis [72] after 9 months. Samples of slurry fluid were collected, filtered using a 0.2 µm filter, and measured for dissolved ammonium concentrations using a Dionex ICS-2000 ion chromatography system (Thermo Scientific) housed at the Environmental Analysis Center at Caltech. Fixed ANME-SRB consortia were separated from the sediment matrix and concentrated following published protocols [5]. Samples were then embedded in Technovit H8100 (Kulzer GmbH, Germany) resin according to published protocols [5, 31] and semi-thin sections (2 µm thickness) were prepared using an Ultracut E microtome (Reichert AG, Austria) which were mounted on Teflon/poly-L-lysine slides (Tekdon Inc., USA). FISH reactions were performed on serial sections (*n* = 30) using Seep1g-1443 and ANME-2b-729 probes as described above, with the omission of 10% SDS to prevent detachment of section from slide (G. Chadwick, personal communication), and slides were imaged and mapped for subsequent nanoSIMS analysis using a Zeiss Elyra PS.1 platform. Sequential sections of each sample were imaged and mapped to identify the section most representative of a section through the center of ANME-SRB consortia. This allowed for the interpretation of spatial patterns correlated with distance from the exterior of the ANME-SRB consortium on the x–y plane as representative of those correlated with the unobserved x–z and y–z planes. After removal of DAPI-Citifuor mounting medium by washing in DI water following published protocols [72], individual wells on the slides were scored with a diamond scribe and cut to fit into the nanoSIMS sample holder (~1 cm diameter) and sputter-coated with 40 nm Au using a Cressington sputter coater. Briefly, nanoSIMS analyses were performed using a Cameca NanoSIMS 50 L housed in Caltech’s Microanalysis Center: 512 × 512 pixel raster images of 20 µm^2^ were collected for ^12^C^–^, ^16^O^–^, ^12^C^14^N^–^, ^15^N^12^C^–^, ^28^Si^–^, and ^32^S^–^ ions by sputtering with a ~1 pA primary Cs^+^ ion beam current with a dwell time of 12–48 ms/pixel. Data were analyzed using look@nanoSIMS [73].

## Results

### 16S rRNA correlation analysis predicts a specific association between ANME-2b and SEEP-SRB1g

**Fig. 1:**
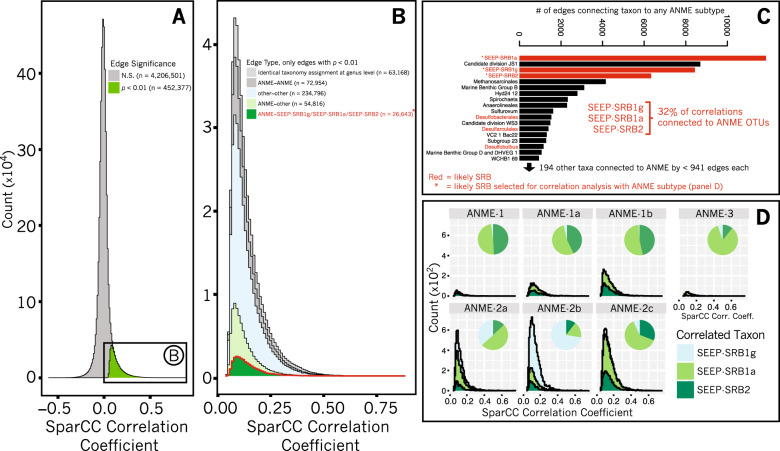
Analysis of SparCC-calculated correlations between 16S rRNA amplicon sequences (OTUs clustered at 99% similarity) from an ecological survey of 310 methane seep sediment samples from seafloor sites off of Costa Rica. A stacked histogram (**a**) illustrates the proportion of correlations deemed significant on the basis of pseudo-*p* values < 0.01 calculated by comparison with 100 bootstrapped correlation tables (see Materials and Methods). Of the correlations with pseudo-*p* values < 0.01, 18% include ANME with a non-ANME taxon (**b**). Significant correlations between OTUs with taxonomy assignments that are identical at the genus level (e.g., two Anaerolinea OTUs) are indicated by identical taxonomy assignment. 32% of correlations between ANME and non-ANME taxa are represented by OTUs assigned to three groups of sulfate-reducing bacteria: SEEP-SRB1g, SEEP-SRB1a, and SEEP-SRB2 (**c**). Stacked histograms of correlations between OTUs assigned to SEEP-SRB1g, SEEP-SRB1a, or SEEP-SRB2 and ANME OTUs, parsed by ANME subtype (**d**), highlights specific associations predicted between ANME-1 and either SEEP-SRB1a or SEEP-SRB2, ANME-2a and SEEP-SRB1a, ANME-2c and SEEP-SRB1a, and between ANME-2b and SEEP-SRB1g.

Correlation analysis applied to 16S rRNA gene amplicon libraries has been frequently used to identify interactions between microorganisms based on the co-occurrence of their 16 S rRNA sequences in different environments or conditions [74–77]. Here, we applied correlation analysis to Illumina 16S rRNA amplicon sequences recovered from Costa Rican methane seep sediments (Supplementary Table 1) to explore partnership specificity between ANME and associated SRB. QIIME processing of amplicon sequences prepared from 310 Costa Rican methane seep sediment and seep carbonate samples yielded 3,052 OTUs after filtering in R. A table of read abundances for these OTUs across the 310 samples was analyzed by SparCC to calculate correlation coefficients and significance for all possible 4,658,878 OTU pairs using 100 bootstraps (Fig. 1a). Of these pairs, 9.7% (452,377) had pseudo-*p* values < 0.01, indicating the coefficients for each of these correlations exceeded that calculated for that same OTU pair in any of the 100 bootstrapped datasets [41]. The taxonomic assignment of the constituent OTUs of correlations with pseudo-*p* values < 0.01 were then inspected, where 18% (81,459) of correlations with pseudo-*p* values < 0.01 describe those involving ANME (Fig. 1b). Of these, 32% occur between ANME and OTUs assigned to three main taxa: *Desulfococcus* sp. (renamed SEEP-SRB1g, see discussion below), SEEP-SRB1a, and SEEP-SRB2 (Fig. 1c). A complete list of significant correlations, their coefficient values, OTU identifiers, and accompanying taxonomy assignments can be found in Supplementary Table 4.

16S rRNA phylogenetic analysis revealed the SILVA-assigned “*Desulfococcus* sp.” OTUs comprise a sister clade to the SEEP-SRB1a that is distinct from cultured *Desulfococcus* sp. (e.g., *D. oleovorans* and *D. multivorans*, see below). We therefore reassigned the *Desulfococcus* OTUs to a new clade we termed SEEP-SRB1g following the naming scheme outlined for seep-associated SRB in Schreiber et al. (e.g., SEEP-SRB1a through -SRB1f) [15]. Furthermore, statistically-significant correlations between OTUs of ANME and SRB taxa suggested that ANME-SRB partnerships in the Costa Rica seep samples could be classified into the following types: ANME-1 with SEEP-SRB1a or SEEP-SRB2, ANME-2a with SEEP-SRB1a, ANME-2b with SEEP-SRB1g, ANME-2c with SEEP-SRB1a or SEEP-SRB2, and ANME-3 with SEEP-SRB1a (Fig. 1d). While physical association between different ANME lineages and Deltaproteobacterial clades SEEP-SRB1a and SEEP-SRB2 had been well-documented [5, 13, 15, 31, 34], members of the SEEP-SRB1g had not previously been identified as a potential syntrophic partner with methanotrophic ANME.

**Fig. 2:**
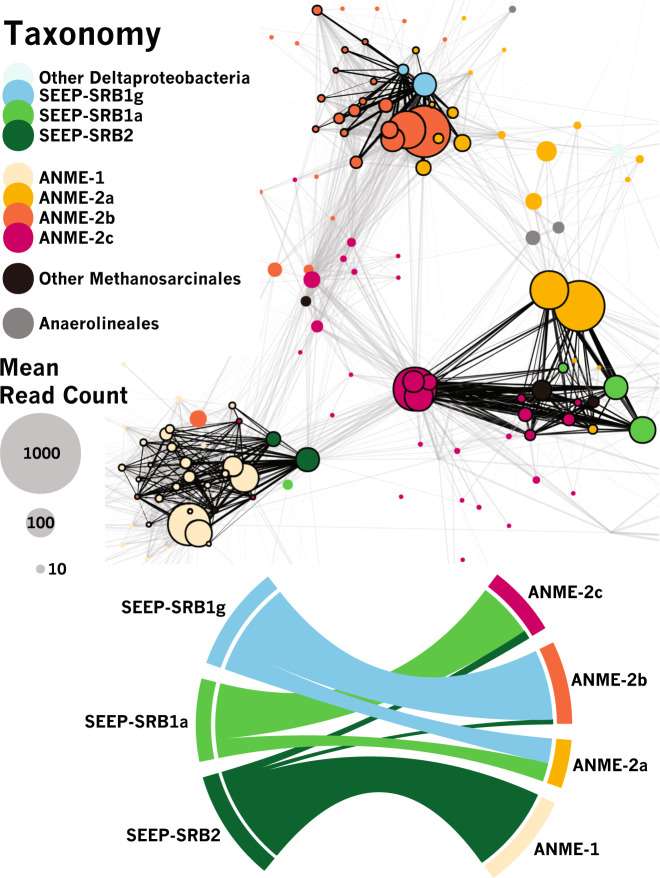
Network analysis of the subset of correlations between OTUs calculated by SparCC [41] that are both significant (pseudo-*p* values <  0.01, 100 bootstraps) and strong (≥99th percentile). Edge length is inversely proportional to correlation strength and is used to visualize the network (top panel) using force-directed methods [78]. Edges are black where they belong to a set of cohesive blocks of nodes [79] and gray otherwise. Chord diagram [80] visualizing ANME-SRB partnership specificity (bottom panel), with band thickness between SRB (left) and ANME (right) proportional to the number of edges between ANME and SRB OTUs within cohesive blocks. Network analysis supports (cf. Fig. 1) previously-undescribed association between ANME-2b and SEEP-SRB1g.

These associations were further examined by detailed network analysis in which the table of correlations with pseudo-*p* values < 0.01 was further filtered to contain only those correlations with coefficients (a measure of correlation strength) in the 99th percentile of all significant correlations. A network diagram in which nodes represent OTUs and edges between nodes represent correlations was constructed with force-directed methods [78], where edge length varied in inverse proportion to correlation strength. A subregion of this network focused on ANME-associated OTUs is presented in Fig. 2. Cohesive blocks, subsets of the graph with greater connectivity to other nodes in the block than to nodes outside [79], were calculated and revealed 3 primary blocks of ANME and SRB OTUs. Visualization of these 3 blocks by a chord diagram [80] further highlighted the taxonomic identity of ANME-SRB OTU pairs in these blocks: ANME-1 or ANME-2c (one OTU with mean read count < 10) and SEEP-SRB2, ANME-2a or ANME-2c and SEEP-SRB1a, and ANME-2b or ANME-2a and SEEP-SRB1g (Fig. 2b). The predicted associations between ANME-2c and SEEP-SRB2 and between ANME-2a and SEEP-SRB1g were relatively more rare than the other associations; only one rare ANME-2c OTU (mean read count < 10) and four uncommon ANME-2a OTUs (mean read count < 100) were predicted between SEEP-SRB2 and SEEP-SRB1g, respectively. Inferred partnership specificity in two of the blocks has been previously corroborated by FISH studies, namely associations between ANME-1 with SEEP-SRB2 [13, 34], ANME-2c with SEEP-SRB1a [5], and ANME-2a with SEEP-SRB1a [15]. The partnership between SEEP-SRB1g and ANME-2b, however, had no precedent, as the only previous FISH descriptions of ANME-2b had placed it with a partner Deltaproteobacterium with taxonomy not known beyond the class level [5, 31].

### Common patterns of association observed in network analysis and in single ANME-SRB consortia

**Fig. 3:**
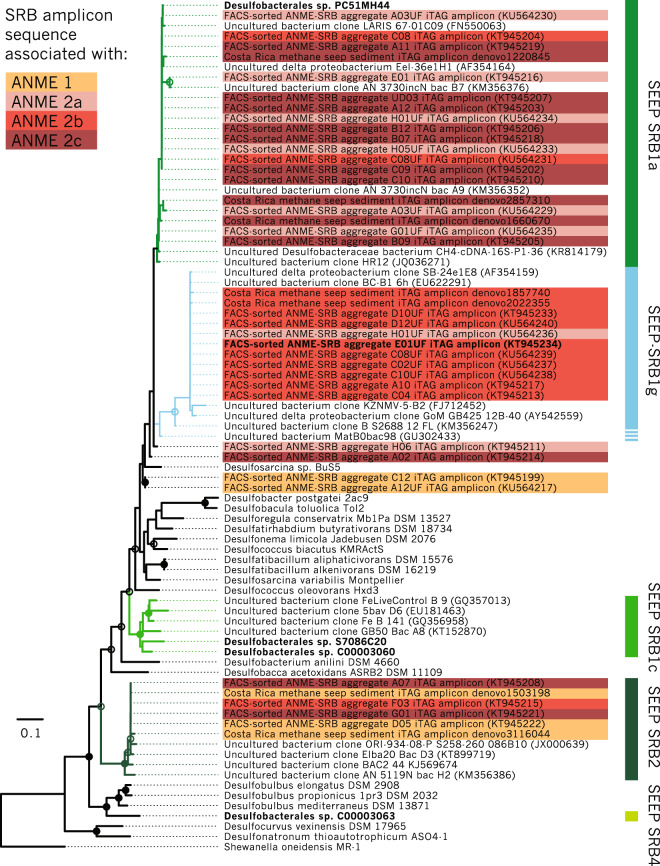
16S rRNA phylogenetic tree of methane seep Deltaproteobacteria and other lineages, including sequences from recovered metagenome-assembled genomes (MAGs) [14], 16S rRNA amplicon sequences from BONCAT-FACS-sorted ANME-SRB consortia [39], 16S rRNA amplicon sequences from this study, and previously published full-length 16S rRNA sequences from clone libraries. Maximum likelihood phylogeny was inferred using 100 bootstraps with >70% or 90% bootstrap support of internal nodes indicated with open or closed circles, respectively. Taxa associated with SRB 16S rRNA amplicon sequences were determined from data in Hatzenpichler et al. [39] (BONCAT-FACS-sorted ANME-SRB consortia), and by network analysis of 16S rRNA amplicon sequences from methane seep samples (cf. Fig. 2). Taxa in bold represent 16S rRNA sequences from MAG bins acquired from methane seep sediments [14] or from BONCAT-FACS-sorted ANME-SRB consortia, including associated 16S rRNA amplicon sequences [39]. The SEEP-SRB1a and -1g clades are operationally defined here by the extent of matches to the respective 16S rRNA FISH probes Seep1a-1441 and Seep1g-1443. Given the low bootstrap values for divergent sequences, the true extent of the SEEP-SRB1g clade is unclear, indicated by the dashed line (cf. Supplementary Fig. 3).

To test if ANME-SRB partnership specificity observed in our correlation analysis of 16S rRNA amplicon sequences from seep samples (Figs. 1, 2) was consistent with data collected from individually-sorted ANME-SRB consortia after BONCAT-FACS [39], we constructed a phylogeny with full-length and amplicon 16S rRNA sequences from ANME-associated SRB including SEEP-SRB1g (Fig. 3; Supplementary Fig. 3). These individual ANME-SRB consortia sorted by BONCAT-FACS were sourced from methane seep sediment samples recovered from Hydrate Ridge off the coast of Oregon and seafloor sites in Santa Monica Basin, California, allowing us to further test whether the ANME-2b—SEEP-SRB1g partnership can be detected in seafloor sites beyond Costa Rica. 16S rRNA amplicon sequences from network analysis of Costa Rica seep sediment samples (Fig. 2) and from BONCAT-FACS sorted consortia from Hydrate Ridge and Santa Monica Basin (Fig. 3; [39]) were then annotated by ANME subtype and identity of associated phylotypes. In the BONCAT-FACS dataset, 8 out of 11 (72%) of the consortia with ANME-2b OTUs had corresponding deltaproteobacterial OTUs that belonged to the SEEP-SRB1g clade (Fig. 3). Similarly, of the Deltaproteobacteria OTU sequences from the BONCAT-FACS sorted consortia affiliated with SEEP-SRB1g 89% (8/9) had ANME-2b as the archaeal partner (Fig. 3). Notably, we found that these SEEP-SRB1g sequences were also highly similar to published full-length 16S rRNA clone library sequences (e.g., NCBI accession AF354159) from seep sediments where ANME-2b phylotypes were also recovered [21]. A *χ*^2^-test for independence was performed on 16S rRNA OTUs recovered from (39) to test the null hypothesis that the presence of a given SRB taxon in a FACS sort is independent of the type of ANME present in the sort. This test demonstrated that the SRB taxon found in a given sort was dependent on the ANME also present in the sort, *χ*^2^ = 30.6 (*d.f*. = 6, *n* = 30), *p* < 0.001. The pattern of association between ANME and SRB OTUs in individual BONCAT-FACS-sorted ANME-SRB consortia thus corroborated the inference from network analysis that ANME-2b and SEEP-SRB1g OTUs exhibit significant partnership specificity. On the basis of amplicon sequence associations found in the BONCAT-FACS sorting dataset collected from sediment samples of Oregon and California seeps as well as those displayed by correlation analysis of amplicons from Costa Rica methane seeps, we designed a set of independent experiments to directly test the hypothesis that ANME-2b form syntrophic partnerships with the previously-undescribed SEEP-SRB1g deltaproteobacteria.

### FISH experiments show SEEP-SRB1g in association with ANME-2b

**Fig. 4:**
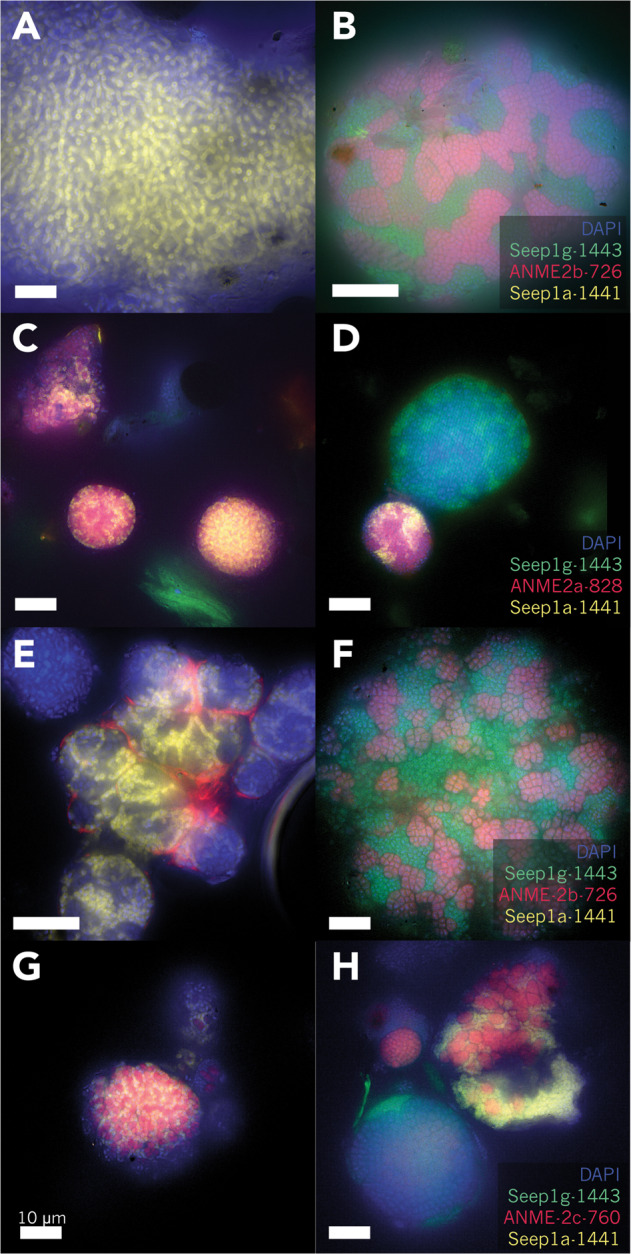
FISH images of ANME-SRB consortia in seep sediment samples using oligonucleotide probes targeting ANME-2b (ANME-2b-726), ANME-2a (ANME-2a-828), or ANME-2c (ANME-2c-760); (in red), a SEEP-SRB1a (Seep1a-1441) probe (in yellow) and a newly-designed probe (Seep1g-1443) targeting the SEEP-SRB1g clade (in green) demonstrating physical association between ANME-2b and SEEP-SRB1g. DAPI counterstain is shown in blue. Seep sediments harboring ANME-2a and ANME-2b (**a**–**d**) host ANME-SRB consortia that are composed of either ANME-2a–SEEP-SRB1a or ANME-2b–SEEP-SRB1g (**b**–**d**). FISH analysis of ANME-SRB consortia from sediments rich in ANME-2c and ANME-2b (**e–h**) documented ANME-SRB consisting of ANME-2b–SEEP-SRB1g or ANME-2c–SEEP-SRB1a partnerships (**f**–**h**); ANME-SRB consortia positively hybridized with the SEEP-SRB1g or SEEP-SRB1a probes were not observed to hybridize with probes targeting ANME-2c (**h**) or ANME-2b (**e**), respectively. In all panels, the scale bar is 10 µm.

Specific oligonucleotide probes were designed and tested for the SEEP-SRB1g clade (Supplementary Fig. 1) and FISH experiments were used to validate the predicted ANME-2b—SEEP-SRB1g partnership. Simultaneous application of FISH probes targeting SEEP-SRB1a, the dominant deltaproteobacterial partner of ANME (Seep1a-1441 [15]), the newly designed SEEP-SRB1g probe (Seep1g-1443, this work), and a probe targeting ANME-2b (ANME-2b-729 [39]) demonstrated that ANME-2b form consortia with SEEP-SRB1g, appearing as large multicellular consortia in seep sediment samples from different localities at Costa Rica methane seep sites (see Supplementary Materials and Methods for site details) that also contain ANME-2a (Fig. 4b, Supplementary Fig. 4) and ANME-2c (Fig. 4f, Supplementary Fig. 5). Results from FISH analysis of >83 consortia from 2 subsamples of seep sediments showed that ANME-2b was not observed in association with SEEP-SRB1a (Fig. 4a, e), and SEEP-SRB1g was not observed in association with ANME-2a (Fig. 4d) or ANME-2c (Fig. 4h) when FISH probes ANME-2a-828 or ANME-2c-760 [20] were substituted for ANME-2b-729. Instead, SEEP-SRB1a was found in consortia with ANME-2a (Fig. 4c) and ANME-2c (Fig. 4g), consistent with previous reports [15]; (Supplementary Fig. 6).

### Genomic potential for N_2_ fixation in sulfate-reducing SEEP-SRB1g deltaproteobacteria

**Fig. 5:**
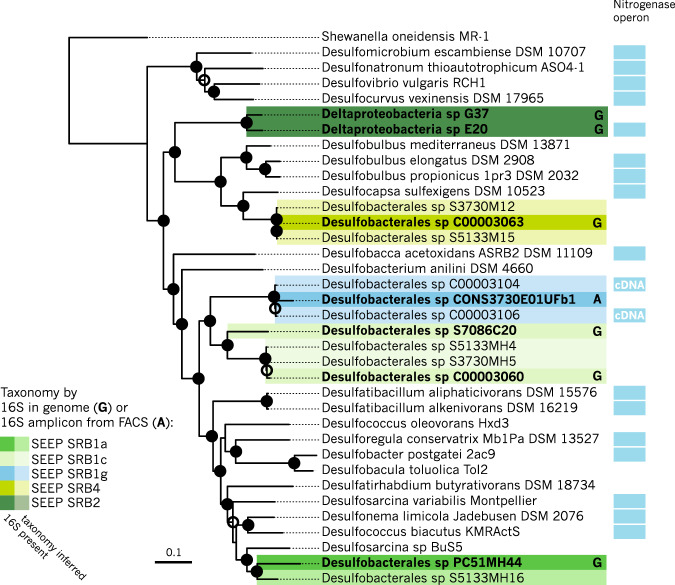
Genome tree of ANME-associated Deltaproteobacteria and related organisms inferred from maximum likelihood methods. Bootstrap support for internal nodes was determined using 100 bootstraps and depicted on the tree as open (>70% bootstrap support) or closed (>90%) circles. Genome bins containing a 16S rRNA gene or an associated 16S rRNA amplicon sequence are highlighted in bold and with a color corresponding to 16 S taxonomy assignment. Inferred taxonomy of genome bins closely related to bins containing 16S rRNA sequences are highlighted in a lighter shade. Genome bins containing the nitrogenase operon are annotated with a blue bar. *nifH* sequences found to be expressed in methane seep sediments in cDNA clone libraries [8] are annotated by “cDNA”. As noted in the text, a search of unpublished SEEP-SRB1a MAGs revealed the presence of highly expressed [8] *nifH* sequences in several unpublished bins (Supplementary Fig. 7).

Given the importance of diazotrophy in the functioning of ANME-SRB syntrophy, we screened metagenome-assembled genome bins (MAGs) of SEEP-SRB1g for the presence of the nitrogenase operon. A genome tree constructed from previously published MAGs from Hydrate Ridge and Santa Monica Basin [14, 39] revealed that two closely related MAGs (Desulfobacterales sp. C00003104, and C00003106) originally classified as belonging to the Seep-SRB1c clade [14] possessed the nitrogenase operon (Fig. 5). These MAGs did not contain 16S rRNA sequences, precluding 16 S rRNA-based taxonomic identification. A more detailed look at these reconstructed genomes revealed that the nitrogenase along with a suite of other genes were unique to this subclade and missing in other SEEP-SRB1c MAGs [14], suggesting they may represent a distinct lineage. In effort to connect these nitrogenase containing SRB MAG’s with representative 16S rRNA sequences, we examined mini-metagenome data from individual BONCAT-FACS sorted ANME-SRB consortia which each contained 16 S rRNA gene sequences for the ANME and bacterial partner [39]. A genome tree containing deltaproteobacterial MAGs [14] and reconstructed deltaproteobacterial genomes from the BONCAT-FACS sorts [39] revealed one SRB genome from a FACS-sorted consortium (Desulfobacterales sp. CONS3730E01UFb1, IMG Genome ID 3300009064) was closely related to the two putative Seep-SRB1c MAGs containing the nitrogenase operon (Fig. 5). The 16S rRNA amplicon sequence (NCBI accession KT945234) associated with this Desulfobacterales sp. CONS3730E01UFb1 genome was used to construct a 16S rRNA phylogeny and confirmed to cluster within the SEEP-SRB1g clade, providing a link between the 16 S rRNA and associated nitrogenase sequences in this lineage (Fig. 3). Given that Desulfobacterales sp. CONS3730E01UFb1, C00003104, and C00003106 genomes appeared highly similar on the genome tree (Fig. 5), we reassigned the previously published Desulfobacterales sp. C00003104 and C00003106 MAGs to the SEEP-SRB1g. Notably, the other 16S rRNA amplicon sequence sampled from the sorted consortium CONS3730E01UF (NCBI accession KT945229) was assigned to ANME-2b [39].

**Fig. 6:**
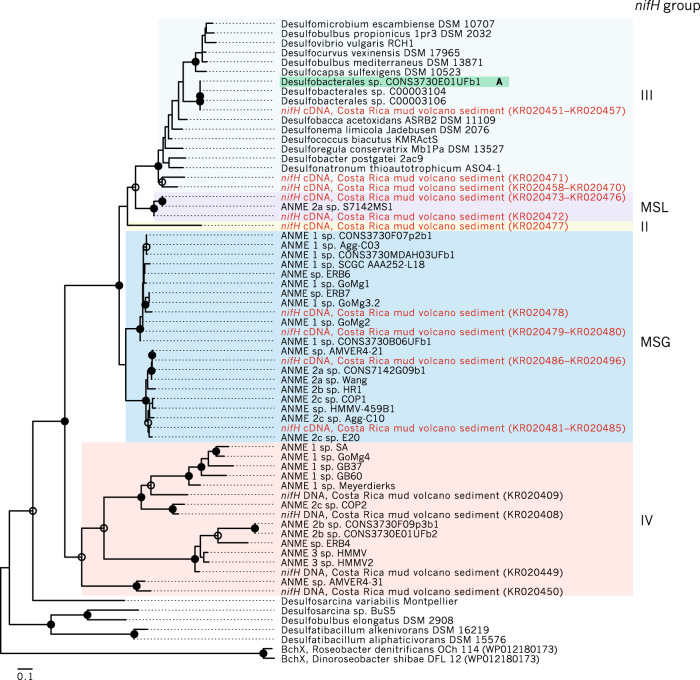
Phylogeny of *nifH* sequences extracted from *nifH* cDNA (red text) and DNA clone libraries [8], from genome bins acquired from methane seep sediments [14], and from other Deltaproteobacteria genomes using a tblastn search with chlorophyllide reductase BchX (WP011566468) as a query. This BchX sequence along with another BchX (WP012180173) were used as an outgroup to root the tree. Phylogeny was inferred by maximum likelihood methods using 100 bootstraps; bootstrap support of internal nodes is illustrated as open or closed circles, indicating >70% or >90% bootstrap support, respectively. *nifH* recovered from the BONCAT-FACS-sorted genome CONS3730E01UFb1, a bin with an accompanying 16S rRNA amplicon sequence placing it within the SEEP-SRB1g, is highlighted in teal. *nifH* groups (sensu Raymond et al. [98]) were assigned by comparison with Dekas et al. [8], and are annotated either by group number or abbreviated as follows: MSL Methanosarcina-like, MSG Methane Seep Group.

The detection of a *nifHDK* operon involved nitrogen fixation (Fig. 5) in the SEEP-SRB1g MAGs was of particular interest as diazotrophy had not previously been an area of focus in the analyses of ANME-associated SRB genomes. A re-analysis of published *nifH* cDNA sequences from methane seep sediments revealed sequences that were nearly identical to the SEEP-SRB1g *nifH* (NCBI accession KR020451-KR020457, [8]) suggesting active transcription of SEEP-SRB1g *nifH* under in situ conditions (Fig. 6, Supplementary File 1). An analysis of published methane seep metaproteomic data [14] also indicated active translation of nitrogenase by SEEP-SRB1g, corroborating evidence from cDNA libraries [8]. In addition, other *nifH* cDNA sequences in this study were found to be identical to nitrogenase sequences occurring in 18 SEEP-SRB1a unpublished metagenome bins (Supplementary Fig. 7) demonstrating that at least some of the syntrophic SEEP-SRB1a SRB partners also possess and actively express *nifH*.

### *nifH* expression in ANME-2b–SEEP-SRB1g consortia visualized by HCR-FISH

**Fig. 7:**
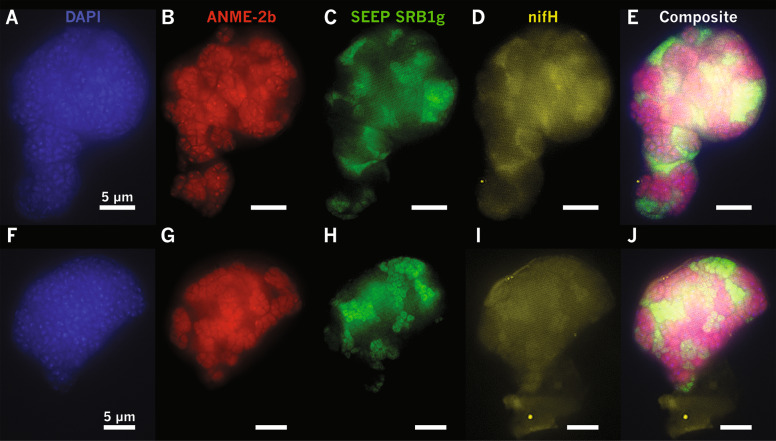
HCR-FISH assays showing in situ expression of *nifH* in SEEP-SRB1g in association with ANME-2b in methane seep sediment incubations, scale bars in all panels are 5 µm. ANME-2b (**b**, **g**) and SEEP-SRB1g (**c**, **h**) cells labeled with FISH probes ANME-2b-729 (in red, [39]) and newly-designed Seep1g-1443 (in green) with DAPI as the DNA counterstain (**a**, **f**). HCR-FISH targeting SEEP-SRB1g *nifH* mRNA (in yellow; Supplementary Table 2) demonstrated active expression of *nifH* transcripts localized to SEEP-SRB1g cells (**d**, **i**, **e**, **j**), supporting the hypothesis of diazotrophy by partner SRB. Control experiments omitting either HCR-FISH initiator probes targeting SEEP-SRB1g *nifH* mRNA or HCR-FISH amplifiers (Supplementary Fig. 8) and colocalization analysis of these control experiments (Supplementary Figs. 9, 10) excluded the possibility that positive signal for SEEP-SRB1g *nifH* was due to bleed-through of fluorescence from Alexa488 bound to the probe targeting SEEP-SRB1g 16S rRNA.

**Fig. 8:**
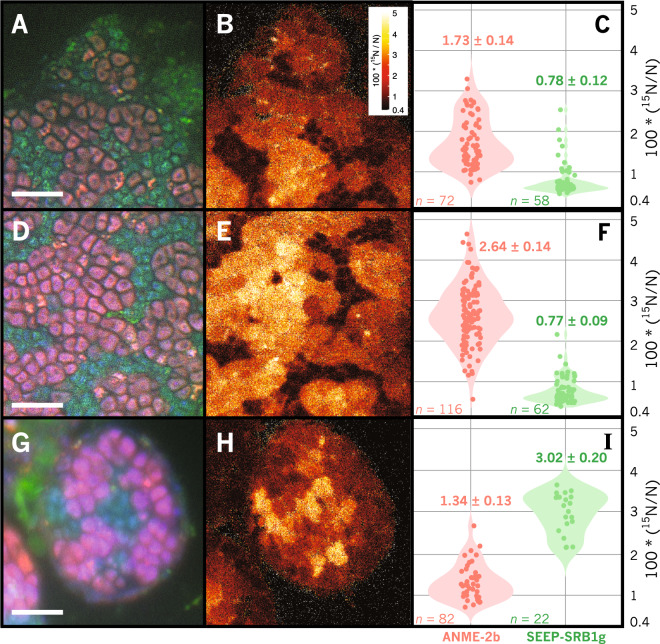
Correlated FISH-nanoSIMS imaging of representative ANME-2b–SEEP-SRB1g consortia demonstrating active diazotrophy by ANME-2b and SEEP-SRB1g cells through ^15^N incorporation from ^15^N_2_. FISH images of ANME-2b (pink) and SEEP-SRB1g (green) are shown in **a**, **d**, **g** and corresponding nanoSIMS ^15^N atom percent values are shown in **b**, **e**, and **h**. Scale bar is 5 µm in **a**, **d**, **g**; raster size in panels B, E, and H is 20 µm^2^. Violin plots (**c**, **f**, **i**) of ^15^N fractional abundance for each type of ROI, representing single ANME-2b or SEEP-SRB1g cells. The number of ROIs measured is indicated by *n* in each panel. Diazotrophic activity in ANME-2b cells appears to be correlated with spatial structure, evidenced by increasing ^15^N enrichment in cells located within consortia interiors (**e**, **f**). SEEP-SRB1g cells are also observed to incorporate ^15^N from ^15^N_2_, and appear to be the dominant diazotroph in the consortium shown in panels G, H, and I, with cellular ^15^N enrichment in SEEP-SRB1g cells greater than that of the paired ANME-2b partner. Abscissa minima set to natural abundance of ^15^N (0.36%).

The dominant role of ANME-2 in nitrogen fixation reported in previous studies [8–10] motivated our examination of whether the sulfate-reducing SEEP-SRB1g partners of ANME-2b were also involved in diazotrophy, either in concert with the ANME-2b partner, or perhaps as the sole diazotroph in this AOM partnership. Using the *nifH* sequences from SEEP-SRB1g, we worked with Molecular Technologies to design a mRNA-targeted probe set to use in whole-cell hybridization chain reaction FISH (HCR-FISH) assays (Supplementary Table 2). HCR-FISH allows for signal amplification and improved signal-to-noise ratio compared to FISH, and has been used in single-cell mRNA expression studies in select microbial studies [81–83]. Prior to this study, however, HCR-FISH had not been applied to visualize gene expression in ANME-SRB consortia from methane seep sediments. In the context of experiments with sediment-hosted ANME-SRB consortia, HCR-FISH provided adequate amplification of the signal to detect expressed mRNA above the inherent background autofluorescence in sediments. Using our HCR-FISH probes targeting SEEP-SRB1g *nifH* mRNA together with the standard 16S rRNA targeted oligonucleotide FISH probes Seep1g-1443 (targeting SEEP-SRB1g) and ANME-2b-729 (targeting ANME-2b), we successfully imaged *nifH* mRNA transcripts by SEEP-SRB1g cells in ANME-2b—SEEP-SRB1g consortia in a sediment AOM microcosm experiment (Fig. 7) in which sediments were incubated in filtered deep-sea water sampled near the seep site. Concentrations of fixed nitrogen species in our incubations were not measured at *t* = 0, but based on independent measurements of porewater ammonium from methane seeps ([NH_4_^+^] = 24–307 µM [10]), we expect some amount (~ µM range) of fixed nitrogen was carried over at the start of our microcosm experiments. We measured dissolved ammonium in the ^15^N_2_ incubations (*n* = 2) ~3 months prior to consortia sampling for nanoSIMS (values ranging from 111–134 µM), and at the time of sampling for nanoSIMS (110 µM to below detection). In this sample, the strongest HCR-FISH *nifH* fluorescence signal from the *nifH* probe set designed to rarget SEEP-SRB1g was observed to in cells identified as the SEEP-SRB1g bacterial partner by 16S rRNA FISH (*n* = 5), with weaker *nifH* fluorescence observed in ANME-2b archaea, but not in co-occurring ANME-2a or -2c consortia. Negative control experiments for the HCR-FISH reaction were also performed. Here, SEEP-SRB1g *nifH* initiator probes were added to the assay, but the fluorescent amplifier hairpins were excluded. In this case, there was no fluorescent signal in either the FISH-stained bacteria or archaeal partners in ANME-2b aggregates indicating that the detected *nifH* HCR-FISH signal (Fig. 7) was not due to native autofluorescence in Seep-SRB1g (Supplementary Fig. 8f–j), nor due to bleed-through of fluorescence from the SEEP-SRB1g 16S rRNA probe. In a second negative control experiment, we excluded the *nifH* initiator probes that bind the mRNA but added the fluorescent amplifier hairpins. This control showed minimal non-specific binding of the hairpins that could be readily differentiated from the positively-hybridized SEEP-SRB1g (Supplementary Fig. 8a–e). Occasionally, highly localized, small spots of fluorescence from the hairpins were observed (Supplementary Fig. 8d) but these spots were primarily localized outside of aggregates and did not align with either bacteria or archaea in consortia (e.g., Fig. 8d). Colocalization image analysis of the control experiments revealed low correlation between FITC (SEEP-SRB1g 16S) or cy3 (ANME-2b 16S) channels with signal in the cy5 (SEEP-SRB1g *nifH*) channel (Supplementary Figs. 9, 10). In contrast, a strong correlation was observed between the FITC and cy5 channels in the HCR-FISH experiment using initiator and amplifiers to detect SEEP-SRB1g *nifH* mRNA expression, producing a linear correlation in a scatterplot of pixel intensities (Supplementary Fig. 11). As noted above, some correlation was also observed between the 16S rRNA ANME-2b signal (cy3) and the HCR-FISH SEEP-SRB1g *nifH* (cy5) channels, indicating that there may be a degree of non-specific binding of the SEEP-SRB1g *nifH* initiator probes to ANME-2b *nifH* mRNA, perhaps due to the conserved nature of nitrogenase sequences. Confirmation of single consortia *nifH* expression in SEEP-SRB1g cells using HCR-FISH corroborated community-level evidence from cDNA libraries (Fig. 6), suggesting the involvement of this sulfate-reducing syntrophic partner in diazotrophy.

### ^15^N_2_ stable isotope probing and FISH-nanoSIMS experiments confirm involvement of SEEP-SRB1g in N_2_-fixation in addition to ANME-2b

To directly test for N_2_ fixation by ANME-2b-associated SEEP-SRB1g, we prepared ^15^N_2_ stable isotope probing incubations of methane seep sediments recovered from a Costa Rica methane seep. These nitrogen-poor sediment incubations were amended with unlabeled methane and ^15^N_2_ and maintained in the laboratory at 10 °C under conditions supporting active sulfate-coupled AOM (see Supplementary Materials and Methods). Sediments with abundant ANME-SRB consortia were sampled after 9 months of incubation and consortia were embedded, sectioned, and analyzed by FISH-nanoSIMS to measure single-cell ^15^N enrichment associated with diazotrophy within ANME-2b—SEEP-SRB1g consortia. Representative ANME-2b—SEEP-SRB1g consortia (*n* = 4) were analyzed by FISH-nanoSIMS and shown to be significantly (~10x) enriched in ^15^N relative to natural abundance values (0.36%; Fig. 8). Among the consortia analyzed, the ^15^N fractional abundance in ANME-2b cells were often higher than that measured in SEEP-SRB1g, with ANME-2b cells on the exterior of an exceptionally large consortium (Fig. 8b, c) featuring ^15^N fractional abundance of 1.73% ± 0.14 (number of ROIs, *n* = 72), significantly enriched relative to that measured in SEEP-SRB1g cells in the exterior, 0.77% ± 0.09 (*n* = 58). In this limited dataset, ANME-2b were observed to fix more nitrogen than their SEEP-SRB1g partners, consistent with previous reports from ANME-2–DSS consortia [8–11]. Notably, however, in one of the 4 ANME-2b—SEEP-SRB1g consortia analyzed, the SEEP-SRB1g cells were more enriched in ^15^N relative to the associated ANME-2b cells, with ANME-2b cells containing 1.34% ± 0.13 ^15^N (*n* = 82) and SEEP-SRB1g containing 3.02% ± 0.20 ^15^N (*n* = 22, Fig. 8i), suggesting that under certain circumstances the sulfate-reducing partner can fix more nitrogen than their ANME-2b partners. Additionally, a gradient in ^15^N enrichment in a large (~250 µm diameter) ANME-2b consortium was observed in which clusters of ANME-2b cells associated with the interior of the consortia, ~10 µm distance from the external environment, were significantly more enriched in ^15^N (Fig. 8f, 2.64% ± 0.14; *n* = 116) relative to ANME-2b clusters near the aggregate exterior (Fig. 8c, 1.73% ± 0.14; *n* = 72). In this consortium, no equivalent ^15^N enrichment gradient was observed in the SEEP-SRB1g partner, with SEEP-SRB1g cells in the exterior containing ^15^N atomic percent values of 0.77% ± 0.09 (*n* = 58) compared with those measured on the interior, 0.78% ± 0.09 (*n* = 62).

## Discussion

The symbiotic relationship between ANME and associated SRB, originally described by Hinrichs [17], Boetius [4], and Orphan [21], has been the focus of extensive study using FISH [5, 7, 13, 15, 25, 26, 29, 34, 35], magneto-FISH [29, 37, 38], and BONCAT-FACS [39], culture-independent techniques that have provided insight into the diversity of partnerships between ANME and SRB. While these fluorescence-based approaches offer direct confirmation of physical association between taxa and are thus useful for characterizing partnership specificity, they are often constrained by sample size and are comparatively lower-throughput than sequencing-based approaches. Next-generation Illumina sequencing of 16S rRNA amplicons offers advantages in terms of throughput and has become a standard approach in molecular microbial ecology. Correlation analysis performed on these large 16S rRNA amplicon datasets can be an effective hypothesis-generating tool for identifying microbial interactions and symbioses in the environment [77], but most studies employing this approach stop short of validating predictions. As correlation analysis of 16S rRNA amplicon data can generate false positives due to the compositional nature of 16 S rRNA amplicon libraries [41, 42, 84], specific correlations predicted between taxa should be corroborated when possible by independent approaches.

In this study, we used correlation analysis of 16S rRNA amplicon sequences from 310 methane seep sediment and carbonate samples on the Costa Rican Margin to identify well-supported (pseudo-*p* values < 0.01) positive correlations between specific OTUs commonly observed in seep ecosystems. Our analysis identified strong correlations between syntrophic partners previously described in the literature, such as that between members of the SEEP-SRB1a and ANME-2a/ or ANME-2c clades and between ANME-1 and SEEP-SRB2 [5, 7, 13, 15, 25, 26, 29, 34, 35], and uncovered previously unrecognized relationships between members of the ANME-2b clade and OTUs affiliated with an uncultured Desulfobacterales lineage, SEEP-SRB1g (Figs. 1–3). We then validated the specificity of the ANME-2b and SEEP-SRB1g association by FISH (Fig. 4).

The specificity of the association between ANME-2b and SEEP-SRB1g appeared to extend beyond Costa Rica methane seeps and is likely a widespread phenomenon, as this association was also recovered from BONCAT-FACS datasets originating from methane seep sites off of Oregon, USA (Hydrate Ridge) and from the Santa Monica Basin, California, USA. Our observations of ANME-2b—SEEP-SRB1g partnership specificity in numerous samples is consistent with published observations of other ANME-SRB partnerships, where consortia composed of specific ANME and SRB clades have been observed in seep ecosystems worldwide [15]. Notably, the syntrophic relationship between ANME-2b and SEEP-SRB1g appears to be specific (Fig. 2), as FISH observations from sediment samples from multiple Costa Rica methane seep sites (Supplementary Table 1) did not show ANME-2b in consortia with other bacteria besides the SEEP-SRB1g (Fig. 4, Supplementary Fig. 6). In contrast, the Desulfobacteraceae SEEP-SRB1a group in these same experiments were found to form associations with both ANME-2a and ANME-2c, indicating that this SRB syntrophic lineage has the capacity to establish partnerships with members of multiple clades of ANME. Members of the diverse ANME-2c family also appeared to display partnership promiscuity in our network analysis, with positive correlations observed between ANME-2c OTUs and both SEEP-SRB1a and SEEP-SRB2 OTUs (Fig. 2). This predicted partnership flexibility in the network analysis was corroborated by our FISH observations of ANME-2c—SEEP-SRB1a consortia (Fig. 4) and additionally by prior reports of ANME-2c in association with SEEP-SRB2 from Guaymas Basin sediments [13]. Taken together, these data suggest that partnership specificity varies among different clades of ANME and SRB, which may be the result of physiological differences and/or molecular compatibility, signal exchange, and recognition among distinct ANME and SRB that shape the degree of specificity between particular ANME and SRB partners, as has been observed in other symbiotic associations [85–87]. The degree of promiscuity or specificity for a given syntrophic partner may be influenced by the co-evolutionary history of each partnership, with some ANME or SRB physiologies requiring obligate association with specific partners. A more detailed examination of the genomes of ANME-2b and SEEP-SRB1g alongside targeted ecophysiological studies may provide clues to the underlying mechanism(s) driving specificity within this ANME-SRB consortia. Comparative investigations with ANME-2a and -2c subgroups may similarly uncover strategies enabling broader partner association, perhaps with preference for a SRB partner shaped by environmental variables rather than through pre-existing co-evolutionary relationships.

An initial genomic screening of SEEP-SRB1g offered some insight into the distinct metabolic capabilities of the SRB partner which may contribute to the association with ANME-2b. The observation of a complete nitrogenase operon in 3 different SEEP-SRB1g genome bins suggested the potential for nitrogen fixation, a phenotype not previously described for ANME-associated SRB (Fig. 5). While previous work on nitrogen utilization by ANME-SRB consortia has focused on diazotrophy performed by ANME-2 [8–10], environmental surveys of seep sediments have noted active expression of nitrogenase typically associated with Deltaproteobacteria [8, 88]. In these studies, the specific microbial taxa associated with the expressed nitrogenase in methane seep sediments were not identified, and based on this community-level analysis, it was not clear whether these putative deltaproteobacterial diazotrophs were involved in AOM syntrophy. A phylogenetic comparison of the *nifH* sequences found in SEEP-SRB1g MAGs with sequences of the expressed deltaproteobacterial-affiliated (i.e., Group III) *nifH* transcripts reported in seep sediments [8] allowed us to link the SEEP-SRB1g syntrophs with a clade of Group III *nifH* sequences that were among the most highly expressed in situ (Figs. 5, 6). FISH-nanoSIMS performed on ^15^N_2_ SIP incubations confirmed the potential for diazotrophic activity in SEEP-SRB1g. Of the 4 ANME-2b—SEEP-SRB1g consortia analyzed by FISH-nanoSIMS, one showed significantly more ^15^N enrichment in the SEEP-SRB1g partner relative to that observed in ANME-2b, while the other 3 displayed higher ^15^N enrichment in ANME-2b cells (Fig. 8). Additional experiments are required to understand the ecological or environmental controls on N_2_ fixation by ANME-2b and SEEP SRB1g; however, our results linking the nitrogenase operon in SEEP-SRB1g MAGs to highly expressed *nifH* transcripts in situ, evidence of *nifH* expression at single-cell level by HCR-FISH, and demonstration of ^15^N_2_ assimilation by FISH-nanoSIMS, all support a role for the SEEP SRB1g in nitrogen fixation as part of methane-oxidizing ANME-2b consortia. Furthermore, the FISH-nanoSIMS ^15^N enrichment patterns within these consortia are suggestive of partner-specific variation in N_2_ fixation either ANME-2b or SEEP-SRB1g, where one partner–ANME-2b or SEEP-SRB1g–fixes nitrogen in excess of the other. We also must consider the fact that the nanoSIMS measures total ^15^N enrichment in cellular biomass, and differences observed ^15^N enrichment between cells can also arise from variation in overall anabolic activity [5], and not exclusively from diazotrophic growth per se. Nevertheless, previous FISH-nanoSIMS data examining ^15^N incorporation from ^15^NH_4_^+^ as a general proxy for anabolic activity revealed that SRB partners to ANME-2b tend to incorporate more ^15^N from supplied ammonium relative to their methanotrophic partners [5], a pattern opposite to that observed in the majority of consortia incubated under a ^15^N_2_ atmosphere. In the light of this previous work, we interpret our nanoSIMS results as indicating that factors beyond taxon-specific differences in nitrogen demand or anabolic activity determine which partner is most diazotrophically active in AOM consortia. Additionally, the observation of nitrogenase in the reconstructed genomes of members of the SEEP-SRB1a clade, representing the most common syntrophic SRB partner of ANME (Supplementary Fig. 7), highlights the possibility that nitrogen fixation may extend to other syntrophic bacterial partners as well and merits further investigation. Re-examination of nitrogen fixation in these partnerships with new FISH probes and nanoSIMS analysis at single-cell resolution will further illuminate the full diversity of diazotrophic activity among ANME-SRB consortia and the associated environmental/ physiological controls.

The factors responsible for determining which partner becomes the most diazotrophically active in ANME-2b—SEEP-SRB1g consortia requires in depth study, but our preliminary data suggest this may be influenced in part by the relative position of ANME-2b or SEEP-SRB1g cells, particularly within large (>50 µm) ANME-2b—SEEP-SRB1g consortia. Previous studies of nitrogen fixation in ANME-SRB consortia found no correlation between consortia size and diazotrophic activity in consortia with diameters <10 µm [10], but larger consortia such as those presented here have not been examined at single-cell resolution within their spatial context in consortia. In addition, aggregates with the morphology observed here, in which ANME-2b cells form multiple sarcinal clusters surrounded by SEEP-SRB1g (Figs. 4b and 8), have not been the specific focus of nanoSIMS analysis but appear to be the common morphotype among ANME-2b—SEEP-SRB1g consortia [31]. The frequency with which this morphotype is observed in ANME-2b—SEEP-SRB1g consortia may be related to the underlying physiology of this specific partnership, which, like other ANME-2 consortia, are assumed to be interacting syntrophically through direct interspecies electron transfer [5]. NanoSIMS analysis of a particularly large ANME-2b—SEEP-SRB1g consortium (~200 µm) with this characteristic morphology (Fig. 8a–f) revealed a gradient in diazotrophic activity in which ANME-2b cells located in the interior of the consortium incorporated far more ^15^N from ^15^N_2_ than ANME-2b cells near the exterior. This pattern may be related to variations in nitrogen supply from the external environment, as similar patterns of nutrient depletion with increasing depth into microbial aggregates have been predicted in modeling studies of nitrate uptake in *Trichodesmium* sp. [89] and directly observed by SIMS in stable isotope probing studies of carbon fixation in biofilm-forming filamentous cyanobacteria [90]. In these examples, modeling and experimental results document declining nitrate or bicarbonate ion availability inwards toward the center of the aggregates resulting from nitrate or bicarbonate consumption. An analogous process may occur in large ANME-2b—SEEP-SRB1g consortia, where cells situated closer to the exterior of the consortium assimilate environmental NH_4_^+^, increasing nitrogen limitation for cells within the consortium core. Interestingly, the single consortium in which the SEEP-SRB1g partner fixed nitrogen in excess of the ANME-2b partner featured SEEP-SRB1g cells in the core of this consortium with ANME-2b cells toward the exterior (Fig. 8). The current nanoSIMS dataset is small and determining the biotic and environmental factors that influence which partner is most diazotrophically active in ANME-2b—SEEP-SRB1g consortia necessitates further study, but a reasonable hypothesis is that the proximity of cells in a given ANME-2b—SEEP-SRB1g consortium relative to the consortium exterior (and NH_4_^+^ availability in the surrounding porewater) influences the spatial patterns of diazotrophic activity by both ANME and SRB in large consortia. The concentration of ammonium in seep porewater can be highly variable over relatively small spatial scales (e.g., between 47 and 299 µM within a single 15 cm-long pushcore [10]), and rates of diazotrophy estimated from laboratory incubations of methane seep sediment samples indicate different threshold concentrations of NH_4_^+^_(aq)_ above which diazotrophy ceases, as low as 25 µM [91] to 100–1000 µM [92–94]. In the large consortia observed here, this threshold [NH_4_^+^_(aq)_] may be crossed within the consortium as NH_4_^+^ is assimilated by cells at the consortium exterior, inducing nitrogen limitation and diazotrophy by ANME or SRB near the consortium core. A simple 1D steady-state reaction-diffusion model of ammonium diffusion and assimilation supports this hypothesis, indicating that for ammonium assimilation rate constants calculated from measurements of bulk methane seep sediment, porewater ammonium concentrations of ~30 µM can produce spatial gradients in diazotrophic activity at length scales of 1–10 µm within AOM consortia (Supplementary File 2), as observed in consortia here (Fig. 8). Ammonium concentrations of 110 µM measured in the SIP incubation from which the ANME-SRB consortium shown in Fig. 8 was sampled suggested that either the rate constant for ammonium assimilation calculated here from bulk assimilation rates is a significant underestimate, or the threshold ammonium concentration for induction of diazotrophy inferred from bulk measurements differs from the threshold relevant for single cells within consortia. Further experimental and modeling work will be necessary to investigate this question. Given the potential importance of diazotrophy for large ANME-SRB consortia and nitrogen cycling in methane seep communities [10, 91], future work should test these hypotheses with ^15^N_2_ incubations under variable [NH_4_^+^_(aq)_].

The observed variation in diazotrophic activity in ANME-2b or SEEP-SRB1g cells may also be the result of phenotypic heterogeneity [95] within the multicellular ANME-2b—SEEP-SRB1g consortia, in which expression of the nitrogenase operon that ANME-2b and SEEP-SRB1g partners both possess is an emergent behavior resulting from the spatial organization of ANME-2b and SEEP-SRB1g cells within the consortium. On the basis of nanoSIMS observations of heterogeneous diazotrophy in clonal *Klebsiella oxytoca* [96] as well as *Crocosphaera watsonii* and *Cyanothece* sp. [97] cultures, phenotypic heterogeneity was inferred to confer selective advantage on microbial communities by enabling rapid response to environmental fluctuations [96] or by facilitating survival in energy-poor environments [97]. Similar heterogeneity in *nif* expression by ANME-2b or SEEP-SRB1g cells may provide partners with resilience against changes in environmental nitrogen supply or expand available niche space through metabolic specialization. Corroborating these observations in diverse ANME-SRB consortia and direct coupling of single-cell mRNA expression with nanoSIMS-acquired ^15^N enrichment would further inform the degree to which relative arrangement of the partners and spatial structure within a consortium plays a significant role in determining the mode of nutrient or electron transfer between partners.

## Conclusions

Here, we present an effective approach to detect novel pairings of microbial symbionts by coupling correlation analysis of 16S rRNA amplicon data with FISH and BONCAT-FACS experiments, going beyond amplicon sequencing-based hypothesis generation to experimental validation of hypothesized partnerships using microscopy and single-cell sorting techniques. Correlation analysis performed on a 16 S rRNA amplicon survey of methane seep sediments near Costa Rica uncovered a novel and highly specific ANME-SRB partnership between ANME-2b archaea and a newly described Desulfobacteraceae-affiliated SEEP-SRB1g bacteria. Partnership specificity was validated by FISH, and further corroborated by 16S rRNA amplicon sequences from BONCAT-FACS-sorted single ANME-SRB consortia from methane seep sediments near Costa Rica, Hydrate Ridge, and Santa Monica Basin in California. Preliminary genomic screening of representatives from SEEP-SRB1g uncovered potential for nitrogen fixation in these genomes. Examination of published *nifH* cDNA clone libraries [8] and transcriptomic data [14] prepared from methane seep sediments demonstrated that SEEP-SRB1g actively expresses *nifH* in situ. The colocalization of positive hybridization signal for *nifH* mRNA using HCR-FISH and SEEP-SRB1g 16S rRNA in ANME-2b—SEEP-SRB1g consortia supported the findings of in situ *nifH* transcription by SEEP-SRB1g. FISH-nanoSIMS analysis of ANME-2b—SEEP-SRB1g consortia recovered from SIP experiments with ^15^N_2_ documented ^15^N incorporation in SEEP-SRB1g cells, confirming that both SEEP-SRB1g and ANME-2b can fix nitrogen. Future work should focus on examining unique aspects of each ANME-SRB syntrophic partnership to improve our understanding of the diversity of microbial symbioses catalyzing the anaerobic oxidation of methane.

## Supplementary information

Supplemental Materials and Methods

Captions for Supplemental Files, Tables, and Figures

Supplemental Table 1

Supplemental Table 2

Supplemental Table 3

Supplemental Table 4

Supplemental Figure 1

Supplemental Figure 2

Supplemental Figure 3

Supplemental Figure 4

Supplemental Figure 5

Supplemental Figure 6

Supplemental Figure 7

Supplemental Figure 8

Supplemental Figure 9

Supplemental Figure 10

Supplemental Figure 11

Supplemental File 1

Supplemental File 2

## References

[1] Knittel K, Boetius A (2009). Anaerobic oxidation of methane: progress with an unknown process. Annu Rev Microbiol.

[2] Reeburgh WS (2007). Oceanic Methane Biogeochemistry. Chem Rev.

[3] Orphan VJ, House CH, Hinrichs K-U, McKeegan KD, DeLong EF (2001). Methane-consuming archaea revealed by directly coupled isotopic and phylogenetic analysis. Science.

[4] Boetius A, Ravenschlag K, Schubert CJ, Rickert D, Widdel F, Gieseke A (2000). A marine microbial consortium apparently mediating anaerobic oxidation of methane. Nature.

[5] McGlynn SE, Chadwick GL, Kempes CP, Orphan VJ (2015). Single cell activity reveals direct electron transfer in methanotrophic consortia. Nature.

[6] Scheller S, Yu H, Chadwick GL, McGlynn SE, Orphan VJ (2016). Artificial electron acceptors decouple archaeal methane oxidation from sulfate reduction. Science.

[7] Wegener G, Krukenberg V, Riedel D, Tegetmeyer HE, Boetius A (2015). Intercellular wiring enables electron transfer between methanotrophic archaea and bacteria. Nature.

[8] Dekas AE, Connon SA, Chadwick GL, Trembath-Reichert E, Orphan VJ (2016). Activity and interactions of methane seep microorganisms assessed by parallel transcription and FISH-NanoSIMS analyses. ISME J.

[9] Dekas AE, Poretsky RS, Orphan VJ (2009). Deep-sea archaea fix and share nitrogen in methane-consuming microbial consortia. Science.

[10] Dekas AE, Chadwick GL, Bowles MW, Joye SB, Orphan VJ (2014). Spatial distribution of nitrogen fixation in methane seep sediment and the role of the ANME archaea. Environ Microbiol.

[11] Orphan VJ, Turk KA, Green AM, House CH (2009). Patterns of 15N assimilation and growth of methanotrophic ANME-2 archaea and sulfate-reducing bacteria within structured syntrophic consortia revealed by FISH-SIMS. Environ Microbiol.

[12] Evans PN, Boyd JA, Leu AO, Woodcroft BJ, Parks DH, Hugenholtz P (2019). An evolving view of methane metabolism in the Archaea. Nat Rev Microbiol.

[13] Krukenberg V, Riedel D, Gruber‐Vodicka HR, Buttigieg PL, Tegetmeyer HE, Boetius A (2018). Gene expression and ultrastructure of meso- and thermophilic methanotrophic consortia. Environ Microbiol.

[14] Skennerton CT, Chourey K, Iyer R, Hettich RL, Tyson GW, Orphan VJ (2017). Methane-fueled syntrophy through extracellular electron transfer: uncovering the genomic traits conserved within diverse bacterial partners of anaerobic methanotrophic archaea. mBio.

[15] Schreiber L, Holler T, Knittel K, Meyerdierks A, Amann R (2010). Identification of the dominant sulfate-reducing bacterial partner of anaerobic methanotrophs of the ANME-2 clade. Environ Microbiol.

[16] Green-Saxena A, Dekas AE, Dalleska NF, Orphan VJ (2014). Nitrate-based niche differentiation by distinct sulfate-reducing bacteria involved in the anaerobic oxidation of methane. ISME J.

[17] Hinrichs K-U, Hayes JM, Sylva SP, Brewer PG, DeLong EF (1999). Methane-consuming archaebacteria in marine sediments. Nature.

[18] Hallam SJ, Girguis PR, Preston CM, Richardson PM, DeLong EF (2003). Identification of methyl coenzyme M Reductase A (mcrA) genes associated with methane-oxidizing archaea. Appl Environ Microbiol.

[19] Michaelis W, Seifert R, Nauhaus K, Treude T, Thiel V, Blumenberg M (2002). Microbial reefs in the black sea fueled by anaerobic oxidation of methane. Science.

[20] Knittel K, Lösekann T, Boetius A, Kort R, Amann R (2005). Diversity and distribution of methanotrophic archaea at cold seeps. Appl Environ Microbiol.

[21] Orphan VJ, Hinrichs K-U, Ussler W, Paull CK, Taylor LT, Sylva SP (2001). Comparative analysis of methane-oxidizing archaea and sulfate-reducing bacteria in anoxic marine sediments. Appl Environ Microbiol.

[22] Orphan VJ, House CH, Hinrichs K-U, McKeegan KD, DeLong EF (2002). Multiple archaeal groups mediate methane oxidation in anoxic cold seep sediments. Proc Natl Acad Sci.

[23] Raghoebarsing AA, Pol A, Pas-Schoonen KT, van de, Smolders AJP, Ettwig KF, Rijpstra WIC (2006). A microbial consortium couples anaerobic methane oxidation to denitrification. Nature.

[24] Haroon MF, Hu S, Shi Y, Imelfort M, Keller J, Hugenholtz P (2013). Anaerobic oxidation of methane coupled to nitrate reduction in a novel archaeal lineage. Nature.

[25] Niemann H, Lösekann T, Beer D, de, Elvert M, Nadalig T, Knittel K (2006). Novel microbial communities of the Haakon Mosby mud volcano and their role as a methane sink. Nature.

[26] Lösekann T, Knittel K, Nadalig T, Fuchs B, Niemann H, Boetius A (2007). Diversity and abundance of aerobic and anaerobic methane oxidizers at the Haakon Mosby Mud Volcano, Barents Sea. Appl Environ Microbiol.

[27] Manz W, Eisenbrecher M, Neu TR, Szewzyk U (1998). Abundance and spatial organization of gram-negative sulfate-reducing bacteria in activated sludge investigated by in situ probing with specific 16S rRNA targeted oligonucleotides. FEMS Microbiol Ecol.

[28] Nauhaus K, Albrecht M, Elvert M, Boetius A, Widdel F (2007). In vitro cell growth of marine archaeal-bacterial consortia during anaerobic oxidation of methane with sulfate. Environ Microbiol.

[29] Pernthaler A, Dekas AE, Brown CT, Goffredi SK, Embaye T, Orphan VJ (2008). Diverse syntrophic partnerships from deep-sea methane vents revealed by direct cell capture and metagenomics. Proc Natl Acad Sci USA.

[30] Vigneron A, Cruaud P, Pignet P, Caprais J-C, Cambon-Bonavita M-A, Godfroy A (2013). Archaeal and anaerobic methane oxidizer communities in the Sonora Margin cold seeps, Guaymas Basin (Gulf of California). ISME J.

[31] McGlynn SE, Chadwick GL, O’Neill A, Mackey M, Thor A, Deerinck TJ (2018). Subgroup characteristics of marine methane-oxidizing ANME-2 archaea and their syntrophic partners as revealed by integrated multimodal analytical microscopy. Appl Environ Microbiol.

[32] Treude T, Krüger M, Boetius A, Jørgensen BB (2005). Environmental control on anaerobic oxidation of methane in the gassy sediments of Eckernförde Bay (German Baltic). Limnol Oceanogr.

[33] Girguis PR, Orphan VJ, Hallam SJ, DeLong EF (2003). Growth and methane oxidation rates of anaerobic methanotrophic archaea in a continuous-flow bioreactor. Appl Environ Microbiol.

[34] Kleindienst S, Ramette A, Amann R, Knittel K (2012). Distribution and in situ abundance of sulfate-reducing bacteria in diverse marine hydrocarbon seep sediments. Environ Microbiol.

[35] Holler T, Widdel F, Knittel K, Amann R, Kellermann MY, Hinrichs K-U (2011). Thermophilic anaerobic oxidation of methane by marine microbial consortia. ISME J.

[36] Loy A, Lehner A, Lee N, Adamczyk J, Meier H, Ernst J (2002). Oligonucleotide Microarray for 16S rRNA Gene-Based Detection of All Recognized Lineages of Sulfate-Reducing Prokaryotes in the Environment. Appl Environ Microbiol.

[37] Trembath-Reichert E, Case DH, Orphan VJ (2016). Characterization of microbial associations with methanotrophic archaea and sulfate-reducing bacteria through statistical comparison of nested Magneto-FISH enrichments. PeerJ.

[38] Trembath-Reichert E, Green-Saxena A, Orphan VJ. Chapter Two—whole cell immunomagnetic enrichment of environmental microbial consortia using rRNA-targeted magneto-FISH. In: DeLong EF (eds). Methods in Enzymology. (Academic Press, San Diego, 2013) pp 21–44. 10.1016/B978-0-12-407863-5.00002-2 24060114

[39] Hatzenpichler R, Connon SA, Goudeau D, Malmstrom RR, Woyke T, Orphan VJ (2016). Visualizing in situ translational activity for identifying and sorting slow-growing archaeal−bacterial consortia. Proc Natl Acad Sci.

[40] Degnan PH, Ochman H (2012). Illumina-based analysis of microbial community diversity. ISME J.

[41] Friedman J, Alm EJ (2012). Inferring correlation networks from genomic survey data. PLOS Comput Biol.

[42] Kurtz ZD, Müller CL, Miraldi ER, Littman DR, Blaser MJ, Bonneau RA (2015). Sparse and compositionally robust inference of microbial ecological networks. PLOS Comput Biol.

[43] Schwager E, Mallick H, Ventz S, Huttenhower C (2017). A Bayesian method for detecting pairwise associations in compositional data. PLOS Comput Biol.

[44] Lima-Mendez G, Faust K, Henry N, Decelle J, Colin S, Carcillo F (2015). Determinants of community structure in the global plankton interactome. Science..

[45] Bohrmann G, Heeschen K, Jung C, Weinrebe W, Baranov B, Cailleau B (2002). Widespread fluid expulsion along the seafloor of the Costa Rica convergent margin. Terra Nova.

[46] Mau S, Sahling H, Rehder G, Suess E, Linke P, Soeding E (2006). Estimates of methane output from mud extrusions at the erosive convergent margin off Costa Rica. Mar Geol.

[47] Sahling H, Masson DG, Ranero CR, Hühnerbach V, Weinrebe W, Klaucke I (2008). Fluid seepage at the continental margin offshore Costa Rica and southern Nicaragua. Geochem Geophys Geosyst.

[48] Glass JB, Yu H, Steele JA, Dawson KS, Sun S, Chourey K (2014). Geochemical, metagenomic and metaproteomic insights into trace metal utilization by methane-oxidizing microbial consortia in sulphidic marine sediments. Environ Microbiol.

[49] Case DH, Pasulka AL, Marlow JJ, Grupe BM, Levin LA, Orphan VJ (2015). Methane seep carbonates host distinct, diverse, and dynamic microbial assemblages. mBio..

[50] Parada AE, Needham DM, Fuhrman JA (2016). Every base matters: assessing small subunit rRNA primers for marine microbiomes with mock communities, time series and global field samples. Environ Microbiol.

[51] Caporaso JG, Kuczynski J, Stombaugh J, Bittinger K, Bushman FD, Costello EK (2010). QIIME allows analysis of high-throughput community sequencing data. Nat Methods.

[52] Mason OU, Case DH, Naehr TH, Lee RW, Thomas RB, Bailey JV (2015). Comparison of archaeal and bacterial diversity in methane seep carbonate nodules and host sediments, Eel River Basin and Hydrate Ridge, USA. Micro Ecol.

[53] Edgar RC (2010). Search and clustering orders of magnitude faster than BLAST. Bioinformatics.

[54] Quast C, Pruesse E, Yilmaz P, Gerken J, Schweer T, Yarza P (2013). The SILVA ribosomal RNA gene database project: improved data processing and web-based tools. Nucleic Acids Res.

[55] Stamatakis A (2014). RAxML version 8: a tool for phylogenetic analysis and post-analysis of large phylogenies. Bioinformatics.

[56] Towns J, Cockerill T, Dahan M, Foster I, Gaither K, Grimshaw A (2014). XSEDE: accelerating scientific discovery. Comput Sci Eng.

[57] Miller MA, Pfeiffer W, Schwartz T. Creating the CIPRES Science Gateway for inference of large phylogenetic trees. In: Proceedings of the 2010 Gateway Computing Environments Workshop (GCE). (San Diego Supercomputing Center, San Diego, 2010) pp 1–8.

[58] Edgar RC (2004). MUSCLE: multiple sequence alignment with high accuracy and high throughput. Nucleic Acids Res.

[59] Eren AM, Esen ÖC, Quince C, Vineis JH, Morrison HG, Sogin ML (2015). Anvi’o: an advanced analysis and visualization platform for ‘omics data. PeerJ.

[60] Campbell BJ, Yu L, Heidelberg JF, Kirchman DL (2011). Activity of abundant and rare bacteria in a coastal ocean. Proc Natl Acad Sci.

[61] Letunic I, Bork P (2019). Interactive Tree Of Life (iTOL) v4: recent updates and new developments. Nucleic Acids Res.

[62] Ludwig W, Strunk O, Westram R, Richter L, Meier H, Yadhukumar (2004). ARB: a software environment for sequence data. Nucleic Acids Res.

[63] Daims H, Stoecker K, Wagner M, Stoecker K, Wagner M. Fluorescence in situ hybridization for the detection of prokaryotes. Mol Microbial Ecol. https://www.taylorfrancis.com/. Accessed 15 Jul 2019.

[64] Glöckner FO, Fuchs BM, Amann R (1999). Bacterioplankton compositions of lakes and oceans: a first comparison based on fluorescence in situ hybridization. Appl Environ Microbiol.

[65] Dirks RM, Pierce NA (2004). Triggered amplification by hybridization chain reaction. Proc Natl Acad Sci.

[66] Choi HMT, Beck VA, Pierce NA (2014). Next-generation in situ hybridization chain reaction: higher gain, lower cost, greater durability. ACS Nano.

[67] Yamaguchi T, Kawakami S, Hatamoto M, Imachi H, Takahashi M, Araki N (2015). In situ DNA-hybridization chain reaction (HCR): a facilitated in situ HCR system for the detection of environmental microorganisms. Environ Microbiol.

[68] Choi HMT, Schwarzkopf M, Fornace ME, Acharya A, Artavanis G, Stegmaier J (2018). Third-generation in situ hybridization chain reaction: multiplexed, quantitative, sensitive, versatile, robust. Development..

[69] Bolte S, Cordelières FP (2006). A guided tour into subcellular colocalization analysis in light microscopy. J Microsc.

[70] Dabundo R, Lehmann MF, Treibergs L, Tobias CR, Altabet MA, Moisander PA, Granger J (2014). The contamination of commercial ^15^N_2_ gas stocks with ^15^N-labeled nitrate and ammonium and consequences for nitrogen fixation measurements. PLoS ONE.

[71] Cline JD (1969). Spectrophotometric determination of hydrogen sulfide in natural waters1. Limnol Oceanogr.

[72] Dekas AE, Orphan VJ. Chapter Twelve—identification of diazotrophic microorganisms in marine sediment via fluorescence in situ hybridization coupled to nanoscale secondary ion mass spectrometry (FISH-NanoSIMS). In: Klotz MG, editor. Methods in enzymology. Academic Press; 2011. p 281–305. 10.1016/B978-0-12-381294-0.00012-2 21185440

[73] Polerecky L, Adam B, Milucka J, Musat N, Vagner T, Kuypers MMM (2012). Look@NanoSIMS-a tool for the analysis of nanoSIMS data in environmental microbiology. Environ Microbiol.

[74] Berry D, Widder S (2014). Deciphering microbial interactions and detecting keystone species with co-occurrence networks. Front Microbiol.

[75] David LA, Maurice CF, Carmody RN, Gootenberg DB, Button JE, Wolfe BE (2014). Diet rapidly and reproducibly alters the human gut microbiome. Nature.

[76] Leone V, Gibbons SM, Martinez K, Hutchison AL, Huang EY, Cham CM (2015). Effects of diurnal variation of gut microbes and high-fat feeding on host circadian clock function and metabolism. Cell Host Microbe.

[77] Ruff SE, Biddle JF, Teske AP, Knittel K, Boetius A, Ramette A (2015). Global dispersion and local diversification of the methane seep microbiome. Proc Natl Acad Sci.

[78] Fruchterman TMJ, Reingold EM (1991). Graph drawing by force-directed placement. Softw Pr Exp.

[79] Moody J, White DR (2003). Structural cohesion and embeddedness: a hierarchical concept of social groups. Am Socio Rev.

[80] Gu Z, Gu L, Eils R, Schlesner M, Brors B (2014). Circlize implements and enhances circular visualization in R. Bioinformatics.

[81] Nikolakakis K, Lehnert E, McFall-Ngai MJ, Ruby EG (2015). Use of hybridization chain reaction-fluorescent in situ hybridization to track gene expression by both partners during initiation of symbiosis. Appl Environ Microbiol.

[82] DePas WH, Starwalt-Lee R, Sambeek LV, Kumar SR, Gradinaru V, Newman DK (2016). Exposing the three-dimensional biogeography and metabolic states of pathogens in cystic fibrosis sputum via hydrogel embedding, clearing, and rRNA Labeling. mBio..

[83] Imachi H, Nobu MK, Nakahara N, Morono Y, Ogawara M, Takaki Y (2020). Isolation of an archaeon at the prokaryote–eukaryote interface. Nature.

[84] Gloor GB, Macklaim JM, Pawlowsky-Glahn V, Egozcue JJ (2017). Microbiome datasets are compositional: and this is not optional. Front Microbiol.

[85] Sampayo EM, Ridgway T, Bongaerts P, Hoegh-Guldberg O (2008). Bleaching susceptibility and mortality of corals are determined by fine-scale differences in symbiont type. Proc Natl Acad Sci.

[86] Parkinson JE, Baumgarten S, Michell CT, Baums IB, LaJeunesse TC, Voolstra CR (2016). Gene expression variation resolves species and individual strains among coral-associated dinoflagellates within the genus symbiodinium. Genome Biol Evol.

[87] Barshis DJ, Ladner JT, Oliver TA, Palumbi SR (2014). Lineage-specific transcriptional profiles of Symbiodinium spp. unaltered by heat stress in a coral host. Mol Biol Evol.

[88] Kapili BJ, Barnett SE, Buckley DH, Dekas AE (2020). Evidence for phylogenetically and catabolically diverse active diazotrophs in deep-sea sediment. ISME J.

[89] Klawonn I, Eichner MJ, Wilson ST, Moradi N, Thamdrup B, Kümmel S (2020). Distinct nitrogen cycling and steep chemical gradients in Trichodesmium colonies. ISME J.

[90] Petroff AP, Wu T-D, Liang B, Mui J, Guerquin-Kern J-L, Vali H (2011). Reaction–diffusion model of nutrient uptake in a biofilm: Theory and experiment. J Theor Biol.

[91] Dekas AE, Fike DA, Chadwick GL, Green‐Saxena A, Fortney J, Connon SA (2018). Widespread nitrogen fixation in sediments from diverse deep-sea sites of elevated carbon loading. Environ Microbiol.

[92] Knapp AN (2012). The sensitivity of marine N_2_ fixation to dissolved inorganic nitrogen. Front Microbiol.

[93] Bertics VJ, Löscher CR, Salonen I, Dale AW, Gier J, Schmitz RA (2013). Occurrence of benthic microbial nitrogen fixation coupled to sulfate reduction in the seasonally hypoxic Eckernförde Bay, Baltic Sea. Biogeosciences.

[94] Gier J, Sommer S, Löscher CR, Dale AW, Schmitz RA, Treude T (2016). Nitrogen fixation in sediments along a depth transect through the Peruvian oxygen minimum zone. Biogeosciences.

[95] Ackermann M (2015). A functional perspective on phenotypic heterogeneity in microorganisms. Nat Rev Microbiol.

[96] Schreiber F, Littmann S, Lavik G, Escrig S, Meibom A, Kuypers MMM (2016). Phenotypic heterogeneity driven by nutrient limitation promotes growth in fluctuating environments. Nat Microbiol.

[97] Masuda T, Inomura K, Takahata N, Shiozaki T, Yuji S (2020). Heterogeneous nitrogen fixation rates confer energetic advantage and expanded ecological niche of unicellular diazotroph populations. Commun Biol.

[98] Raymond J, Siefert JL, Staples CR, Blankenship RE (2004). The natural history of nitrogen fixation. Mol Biol Evol.

