# Supplementary material for: Experimentally-validated correlation analysis reveals new anaerobic methane oxidation partnerships with consortium-level heterogeneity in diazotrophy
Source: ISME J. 2020 Oct 15;15(2):377–96. doi: 10.1038/s41396-020-00757-1 (PMC8027057; doi:10.1038/s41396-020-00757-1)
Supplement: Supplementary file 2 — Captions for Supplemental Files, Tables, and Figures [file 41396_2020_757_MOESM2_ESM.docx]

Supplemental Figure 2. Krona chart depicting relative abundance of taxa in Costa Rica seep sediment sample #10073 (Fig. 7) as measured by 16S rRNA amplicon sequencing.

Supplemental Figure 3. Krona chart depicting relative abundance of taxa in Costa Rica seep sediment sample #9279 (Fig. 4) as measured by 16S rRNA amplicon sequencing.

Supplemental Figure 4. Krona chart depicting relative abundance of taxa in Costa Rica seep sediment sample #9112 (Fig. 4) as measured by 16S rRNA amplicon sequencing.

Supplemental Table 1. Samples of methane seep sediment used in this study to produce 16S rRNA amplicon libraries.

Supplemental Table 2. Newly-designed FISH probe (Seep1g-1443) and *nifH* mRNA HCR-FISH probe for labeling ANME-associated members of SEEP-SRB1g or SEEP-SRB1g *nifH* transcripts, respectively. Bolded sequence is complementary to HCR-FISH amplifier B1; nonbolded sequence is complementary to SEEP-SRB1g 16S rRNA or *nifH* RNA. Matches determined by comparison with ARB/SILVA SSU release 128 [54].

Supplemental Table 3. Stable isotope probing incubation conditions, sample sources and sulfide concentration measurements as a proxy for sulfate reduction activity.

Supplemental Table 4. SparCC-calculated correlations (pseudo-*p* < 0.01) between OTUs, detailing coefficients, OTU identifiers, and taxonomy assignments.

Supplemental File 1. FASTA file containing the translated amino acid sequences for *nifH* included in Figure 6 in select ANME and SRB genomes (Chadwick, et al., *in prep*) and transcripts [8].

Supplemental File 2. HTML-formatted Jupyter Notebook describing the 1D steady-state reaction-diffusion model calculating the depth inside of an ANME-SRB consortia at which ammonium assimilation depletes ammonium concentrations below the measured threshold for induction of diazotrophic activity in methane seep sediments.
