# Supplementary material for: Experimentally-validated correlation analysis reveals new anaerobic methane oxidation partnerships with consortium-level heterogeneity in diazotrophy
Source: ISME J. 2020 Oct 15;15(2):377–96. doi: 10.1038/s41396-020-00757-1 (PMC8027057; doi:10.1038/s41396-020-00757-1)
Supplement: Supplementary file 7 — Supplemental Figure 1 [file 41396_2020_757_MOESM7_ESM.pdf]

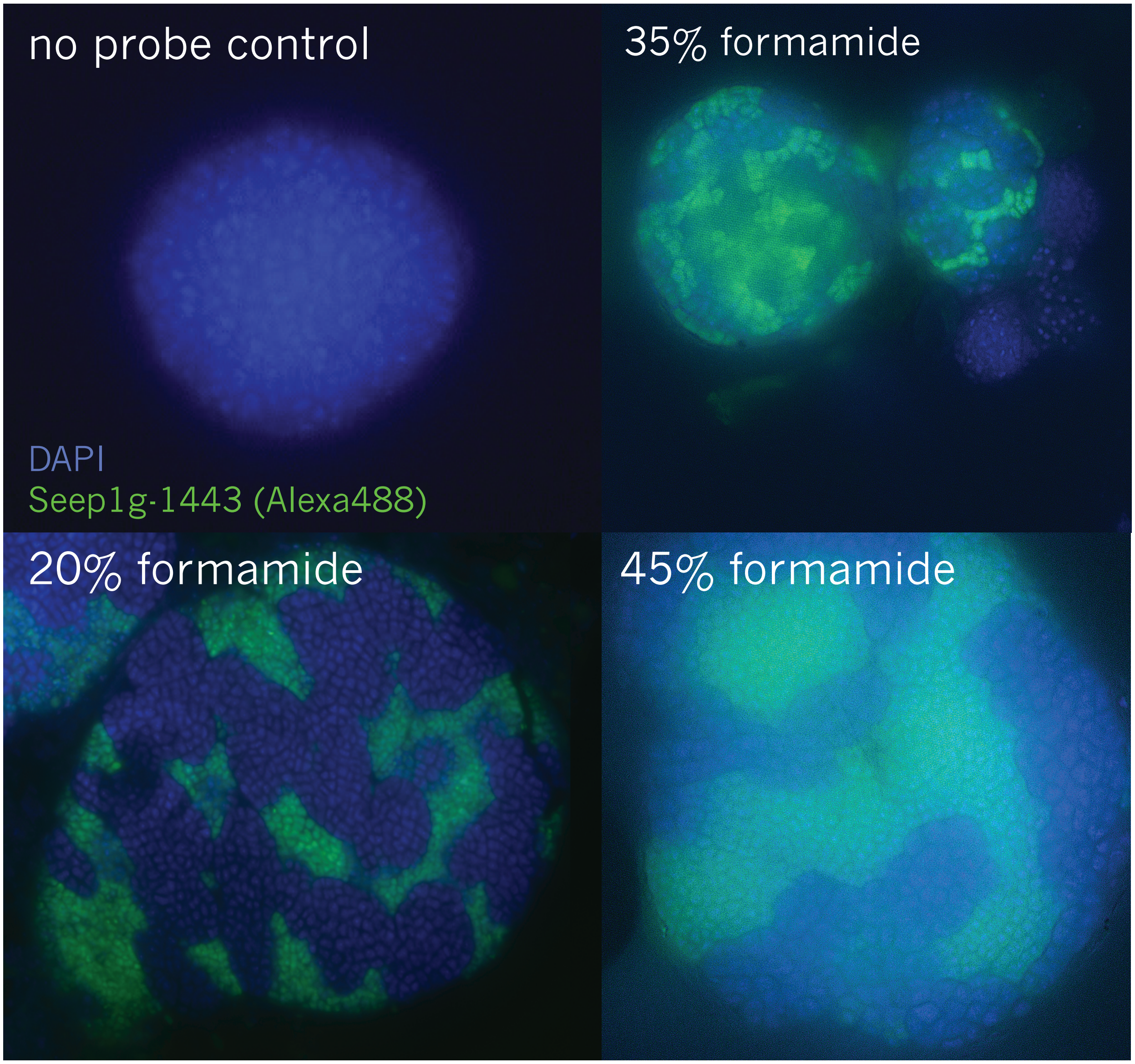

Supplemental Figure 1. Optimization of the newly designed Seep1g-1443 probe by FISH hybridization of ANME-2b—SEEP-SRB1g consortia at a range of formamide concentrations.
