# Supplementary material for: Experimentally-validated correlation analysis reveals new anaerobic methane oxidation partnerships with consortium-level heterogeneity in diazotrophy
Source: ISME J. 2020 Oct 15;15(2):377–96. doi: 10.1038/s41396-020-00757-1 (PMC8027057; doi:10.1038/s41396-020-00757-1)
Supplement: Supplementary file 9 — Supplemental Figure 3 [file 41396_2020_757_MOESM9_ESM.pdf]

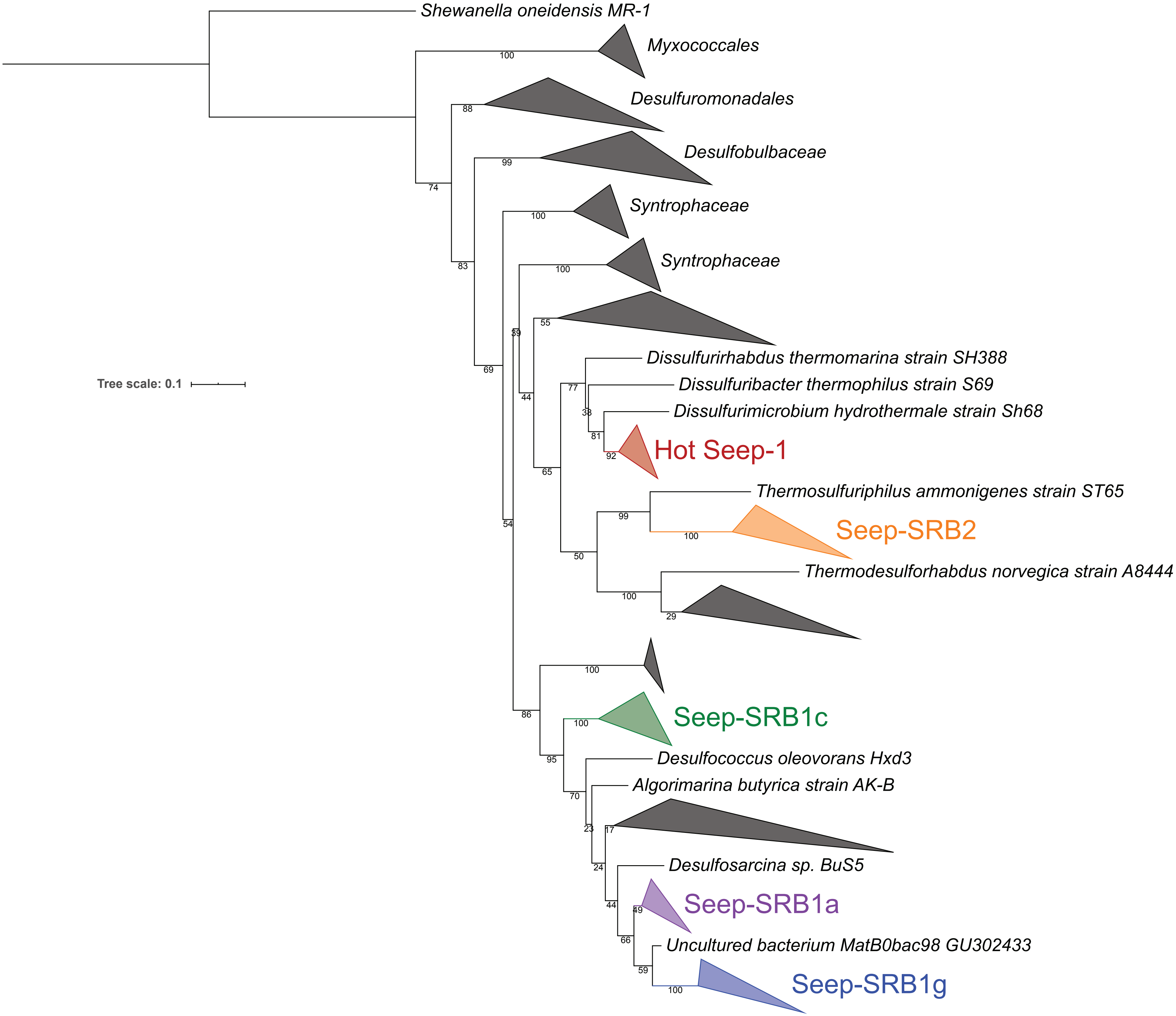

Supplemental Figure 3. 16S rRNA phylogeny inferred from maximum-likelihood methods using only full-length 16S rRNA sequences. Tree topology shown here is congruent with the phylogeny shown in Figure 3 constructed using a mix of shorter 16S rRNA amplicon and full-length 16S sequences.
