# Supplementary material for: Experimentally-validated correlation analysis reveals new anaerobic methane oxidation partnerships with consortium-level heterogeneity in diazotrophy
Source: ISME J. 2020 Oct 15;15(2):377–96. doi: 10.1038/s41396-020-00757-1 (PMC8027057; doi:10.1038/s41396-020-00757-1)
Supplement: Supplementary file 12 — Supplemental Figure 6 [file 41396_2020_757_MOESM12_ESM.pdf]

SEEP-SRB1g, ANME-2b, SEEP-SRB1a

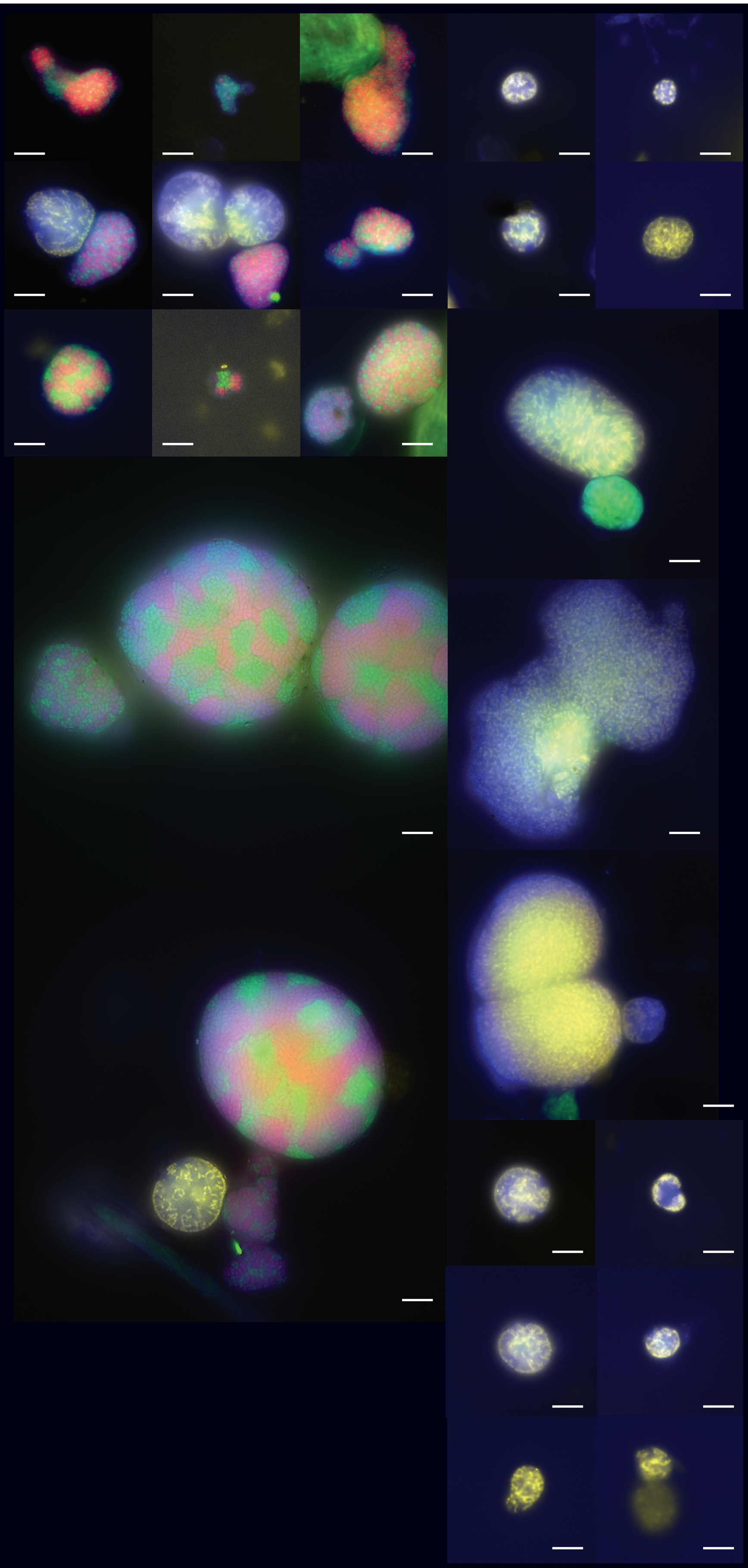

	SEEP-SRB1g	SEEP-SRB1a
ANME-2b	18	0
DAPI only	0	19

SEEP-SRB1g, ANME-2a, SEEP-SRB1a

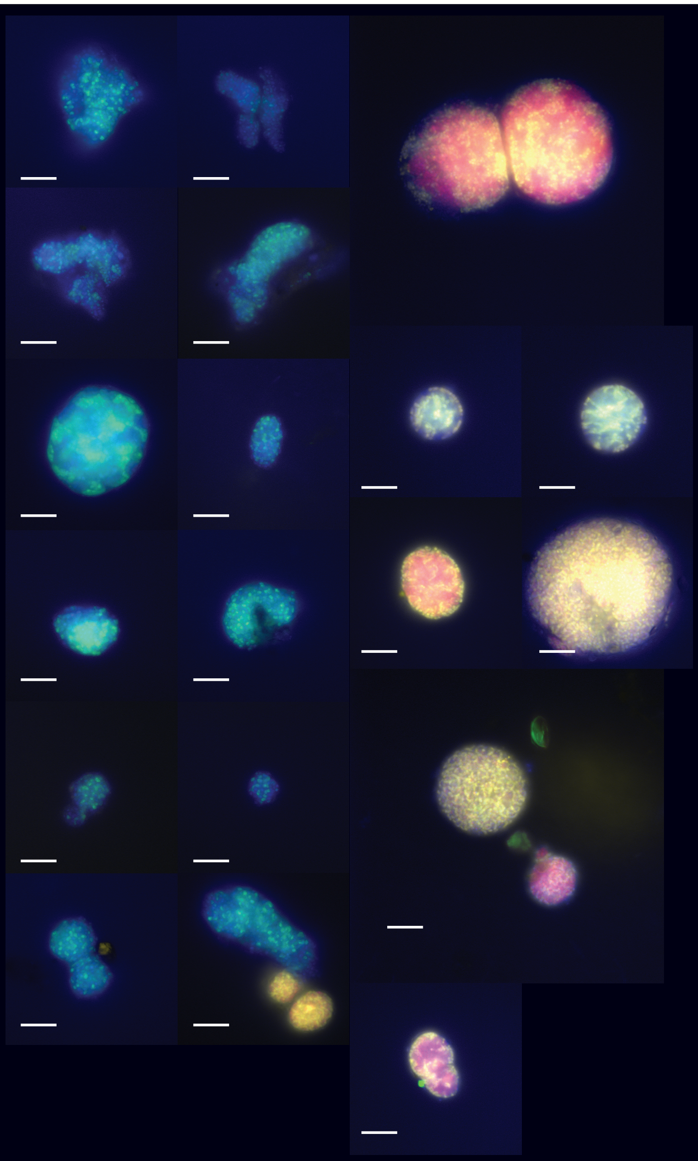

	SEEP-SRB1g	SEEP-SRB1a
ANME-2a	0	7
DAPI only	13	2

Supplemental Figure 6. Quantification of ANME-SRB partnership pairings in Costa Rica seep sediment sample #9279 using 16S rRNA FISH experiments, using probes Seep1g-1443, ANME-2b-729, and SEEP-SRB1a (left panel) and a complementary experiment (right panel) in which the ANME-2b probe was exchanged for ANME-2a-828. DAPI signal appears as blue in all images. Scale bar (white) for all images is 10 µm. Tabulation of consortia comprised of each pairing is presented at the bottom of each column, showing highly specific pairing between ANME-2b and SEEP-SRB1g.
