# Supplementary material for: Experimentally-validated correlation analysis reveals new anaerobic methane oxidation partnerships with consortium-level heterogeneity in diazotrophy
Source: ISME J. 2020 Oct 15;15(2):377–96. doi: 10.1038/s41396-020-00757-1 (PMC8027057; doi:10.1038/s41396-020-00757-1)
Supplement: Supplementary file 13 — Supplemental Figure 7 [file 41396_2020_757_MOESM13_ESM.pdf]

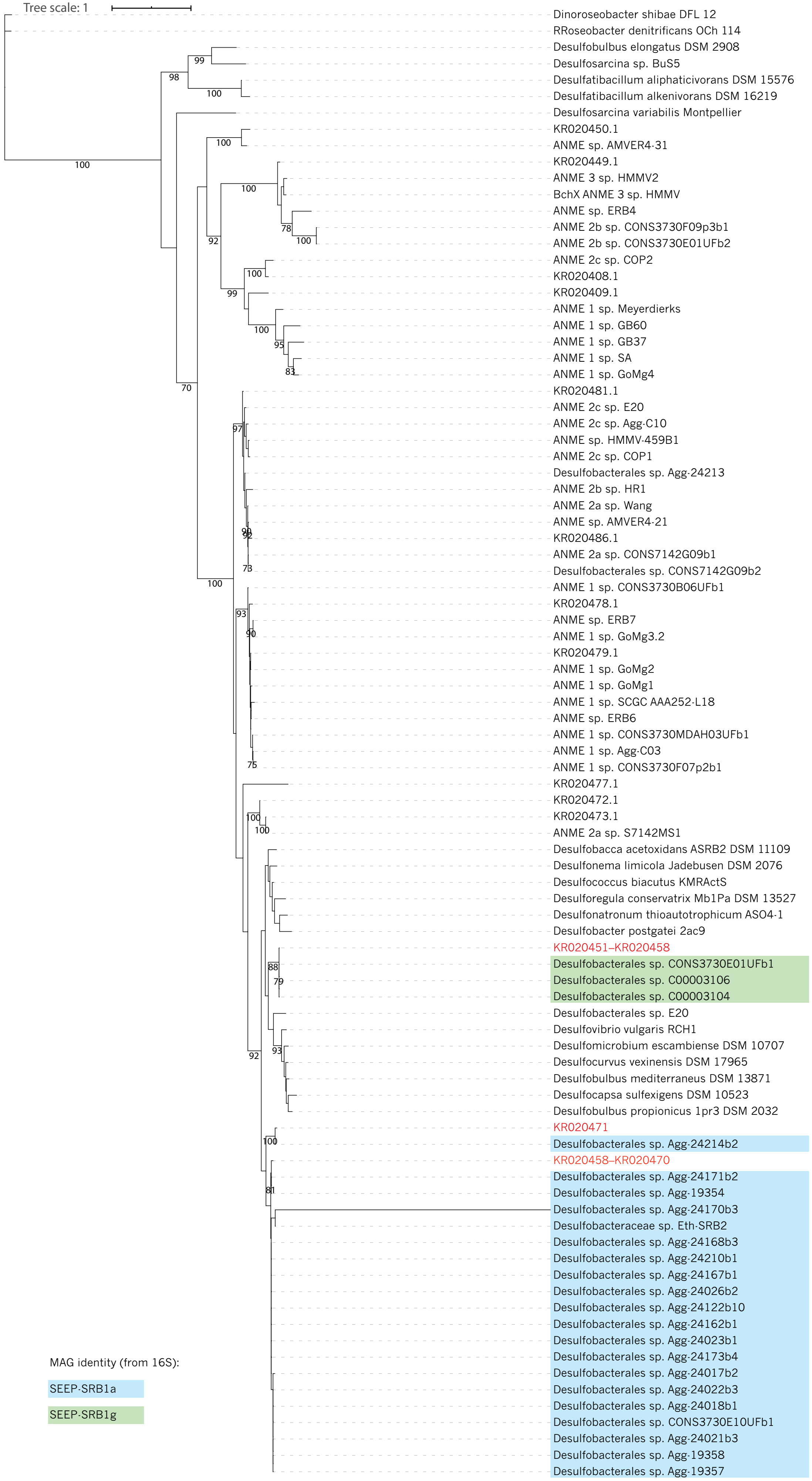

Supplemental Figure 7. Extended nifH tree including unpublished SEEP-SRB1a MAGs possessing nifH sequences nearly identical to some recovered in environmental cDNA libraries (Dekas, et al. 2016).
