# Supplementary material for: Experimentally-validated correlation analysis reveals new anaerobic methane oxidation partnerships with consortium-level heterogeneity in diazotrophy
Source: ISME J. 2020 Oct 15;15(2):377–96. doi: 10.1038/s41396-020-00757-1 (PMC8027057; doi:10.1038/s41396-020-00757-1)
Supplement: Supplementary file 14 — Supplemental Figure 8 [file 41396_2020_757_MOESM14_ESM.pdf]

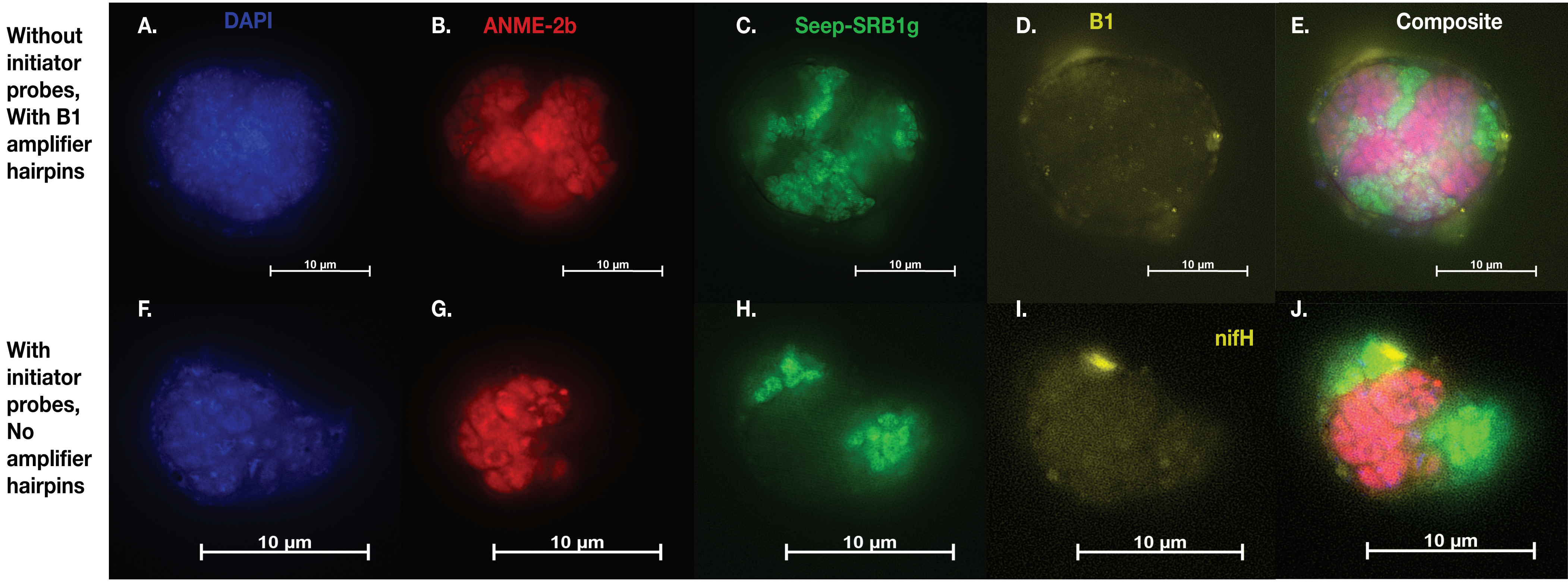

Supplementary Figure 8. Representative images for the negative control experiments for visualizing mRNA expression by HCR-FISH. (A-E) Images of ANME-2b/Seep-1g aggregates in the DAPI, TRITC, FITC and cy5 channels as well as the composite of images from all the channels. These images were from an experiment without nifH-targeted initiator probes but with just B1 fluorescent hairpins in order to visualize potential non-specific binding of B1 hairpins. (F-J) These images were from an experiment with nifH-targeted initiator probes but without B1 fluorescent hairpins in order to visualize the background fluoresence in the the samples.
