# Supplementary material for: Experimentally-validated correlation analysis reveals new anaerobic methane oxidation partnerships with consortium-level heterogeneity in diazotrophy
Source: ISME J. 2020 Oct 15;15(2):377–96. doi: 10.1038/s41396-020-00757-1 (PMC8027057; doi:10.1038/s41396-020-00757-1)
Supplement: Supplementary file 15 — Supplemental Figure 9 [file 41396_2020_757_MOESM15_ESM.pdf]

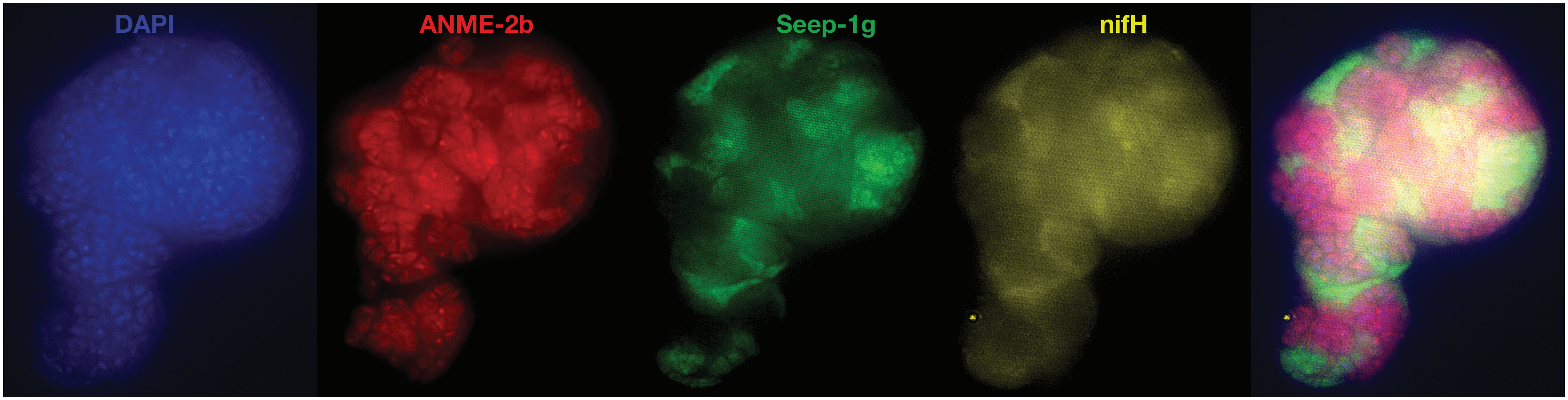

Correlation between ANME-2b and Seep-1g fluorescent signal

Pearson's correlation coefficient, **PC=0.661**, p-value-100%, Costes' method

Manders' correlation coefficients, after thresholding, **M1=0.859, M2=0.552**

Correlation between Seep-1g and nifH fluorescent signal

Pearson's correlation coefficient, **PC=0.893**, p-value=100%, Costes method

Manders' correlation coefficients, after thresholding, **M1=0.964, M2=0.733**

Correlation between ANME-2b and nifH fluorescent signal

Pearson's correlation coefficient, **PC=0.837**, p-value =Costes' method

Manders' correlation coefficients, after thresholding, **M1=0.805, M2=0.905**

a. Scatterplots of pixel intensities

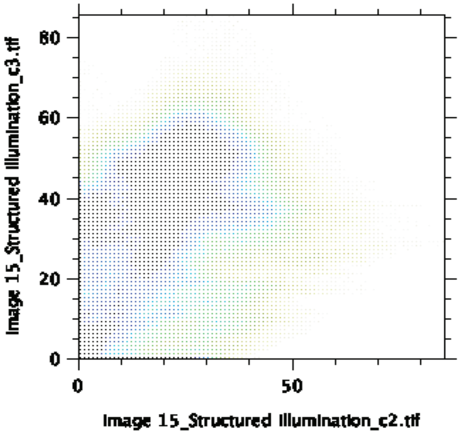

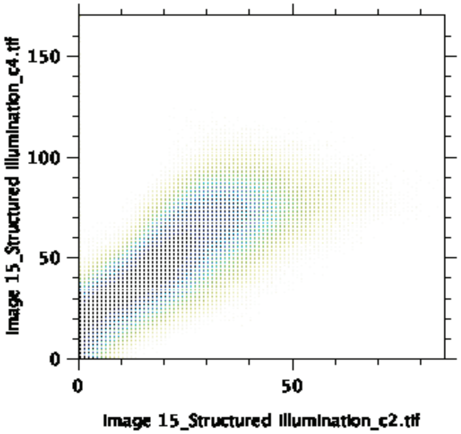

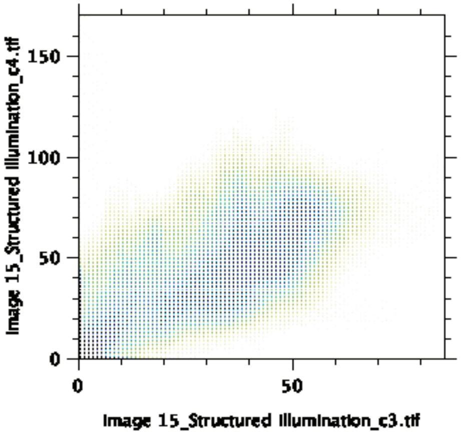

Supplementary Figure 9. Colocalization analysis of HCR-FISH experimental data to investigate nitrogenase expression by nifH targeted probes. ANME-2b is stained in the cy3 channel, Seep-1g in the FITC channel and the B1 amplifiers binding nifH initiator probes are visualized in the cy5 channel a. Scatterplots of pixel intensities of the FITC, cy3 and cy5 channel suggest that there is correlation between the Seep-1g signal and nifH signal, as well as correlation between ANME-2b and the nifH signal. However, there appears to be more noise in the latter, rather than just a linear correlation between the ANME-2b and nifH signals. An equally high PC between between Seep-1g and nifH, and ANME-2b and nifH is suggestive of nifH expression in ANME-2b as well. This is not an observation that is visually obvious and the lower intensity signal could come from nifH probes designed to target Seep-1g, binding ANME-2b nifH with lower efficiency. The Manders' coefficients suggest that almost all of the Seep-1g cells correlate with nifH signal while the same is not true of the colocalization of nifH signal with ANME-2b.
