# Supplementary material for: Experimentally-validated correlation analysis reveals new anaerobic methane oxidation partnerships with consortium-level heterogeneity in diazotrophy
Source: ISME J. 2020 Oct 15;15(2):377–96. doi: 10.1038/s41396-020-00757-1 (PMC8027057; doi:10.1038/s41396-020-00757-1)
Supplement: Supplementary file 16 — Supplemental Figure 10 [file 41396_2020_757_MOESM16_ESM.pdf]

HCR-FISH control experiment: With initiator probes, No amplifier hairpins

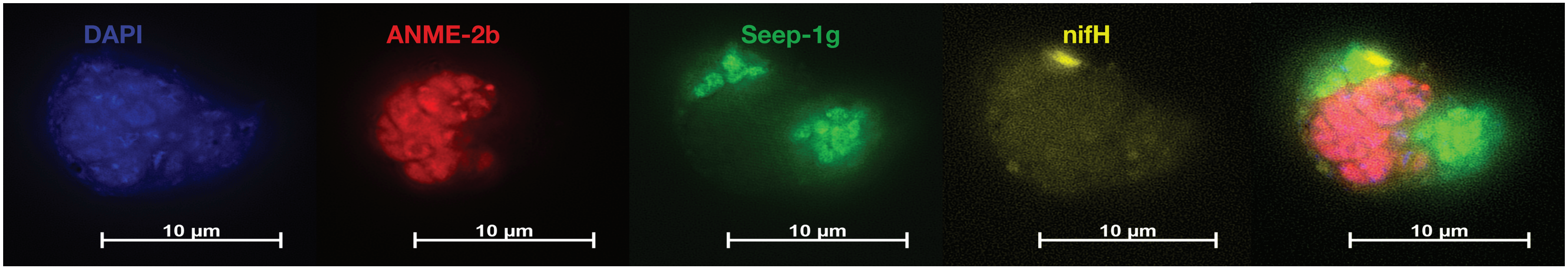

Correlation between ANME-2b and Seep-1g fluorescent signal

Pearson's correlation coefficient, **PC=0.165**, p-value-100%, Costes' method

Manders' correlation coefficients, after thresholding, **M1=0.022, M2=0.008**

Correlation between Seep-1g and nifH fluorescent signal

Pearson's correlation coefficient, **PC=0.541**, p-value=100%, Costes method

Manders' correlation coefficients, after thresholding, **M1=0.891, M2=0.163**

Correlation between ANME-2b and nifH fluorescent signal

Pearson's correlation coefficient, **PC=0.521**, p-value =Costes' method

Manders' correlation coefficients, after thresholding, **M1=0.958, M2=0.39**

a. Scatterplots of pixel intensities

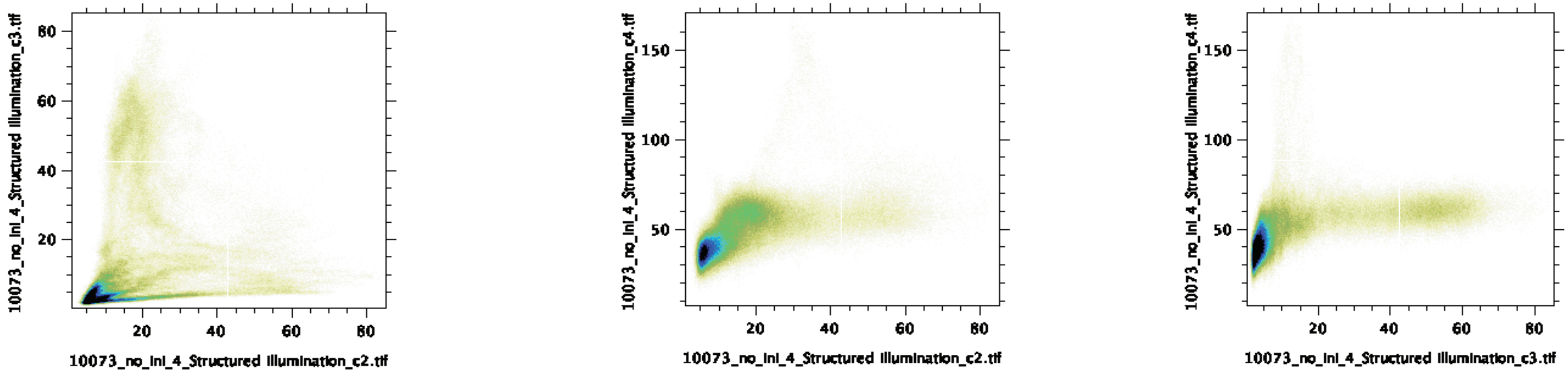

Supplementary Figure 10. Colocalization analysis of HCR-FISH control experiment with initiator probes and without amplifier hairpins. ANME-2b is stained in the cy3 channel, Seep-1g in the FITC channel. No dyes fluorescing in the cy5 channel are present in this experiment a. Scatterplots of pixel intensities of the FITC, cy3 and cy5 channel suggest there is some bleed through in both the cy3 and FITC channels.Neither the Pearson's correlation coefficient which measures covariance between the channels in proportion to their standard deviation nor Manders' correlation coefficients M1 and M2, which better correct for differences in intensity, are high enough to indicate significant cross-correlation.
