# Supplementary material for: Experimentally-validated correlation analysis reveals new anaerobic methane oxidation partnerships with consortium-level heterogeneity in diazotrophy
Source: ISME J. 2020 Oct 15;15(2):377–96. doi: 10.1038/s41396-020-00757-1 (PMC8027057; doi:10.1038/s41396-020-00757-1)
Supplement: Supplementary file 17 — Supplemental Figure 11 [file 41396_2020_757_MOESM17_ESM.pdf]

HCR-FISH control experiment: Without initiator probes, with amplifier hairpins

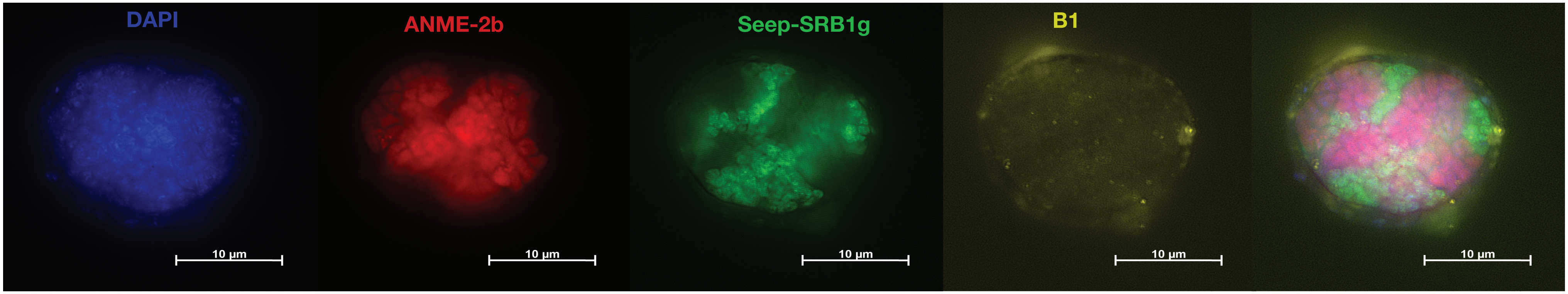

Correlation between ANME-2b and Seep-1g fluorescent signal

Pearson's correlation coefficient, **PC=0.666**, p-value-100%, Costes' method

Manders' correlation coefficients, after thresholding, **M1=0.801, M2=0.601**

Correlation between Seep-1g and nifH fluorescent signal

Pearson's correlation coefficient, **PC=0.332**, p-value=100%, Costes method

Manders' correlation coefficients, after thresholding, **M1=0.002, M2=0.127**

Correlation between ANME-2b and nifH fluorescent signal

Pearson's correlation coefficient, **PC=0.267**, p-value =Costes' method

Manders' correlation coefficients, after thresholding, **M1=0.02, M2=0.221**

a. Scatterplots of pixel intensities

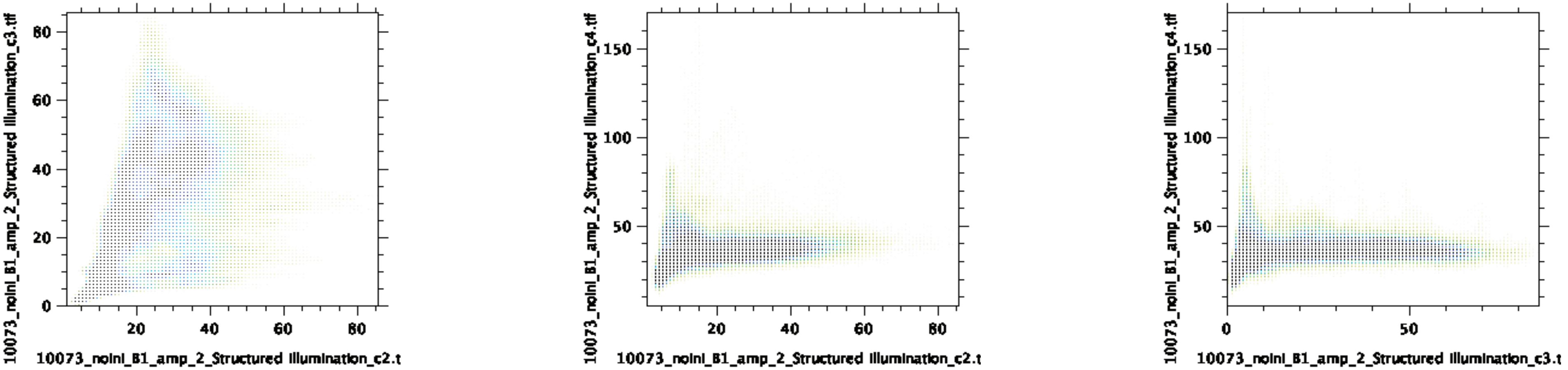

Supplementary Figure 11. Colocalization analysis of HCR-FISH control experiment without initiator probes and with B1 amplifier hairpins. ANME-2b is stained in the cy3 channel, Seep-1g in the FITC channel and the B1 amplifiers, without the initiator probes are visualized in the cy5 channel a. Scatterplots of pixel intensities of the FITC, cy3 and cy5 channel suggest there is some bleed through in both the cy3 and FITC channels. Similar to the no amplifier control, neither the Pearson's correlation coefficient nor M1 and M2 are high enough to indicate significant cross-correlation.
