# Supplementary material for: Experimentally-validated correlation analysis reveals new anaerobic methane oxidation partnerships with consortium-level heterogeneity in diazotrophy
Source: ISME J. 2020 Oct 15;15(2):377–96. doi: 10.1038/s41396-020-00757-1 (PMC8027057; doi:10.1038/s41396-020-00757-1)
Supplement: Supplementary file 19 — Supplemental File 2 [file 41396_2020_757_MOESM19_ESM.html]

diffusion\_model


# Supplemental File 2¶

In [1]:

```
import numpy as np
from numpy import ma
import matplotlib
import matplotlib.pyplot as plt
from matplotlib import cm
```

We hypothesize that preference for ammonium assimilation over nitrogen fixation consumed ammonium diffusing inwards into the ANME-SRB consortia shown in Figure 8, forcing cells in the consortium interior to fix additional nitrogen from $^{15}N\_2$ to compensate for the decrease in N source from ammonium uptake. Here, we model ammonium diffusion into an ANME-SRB consortium and simultaneous ammonium assimilation as a classic 1D diffusion problem with boundary conditions describing continuous input at a fixed location (x = 0) with decay:

\begin{align}
\frac{\partial C}{\partial t} = D\frac{\partial^2 C}{\partial t^2} -kC
\end{align}

where $C$ is the concentration of ammonium at time $t$, $D$ is the diffusivity for dissolved ammonium, and $k$ is the first-order rate constant for ammonium assimilation.

Solving this differential equation at steady state $\left(\frac{\partial C}{\partial t} = 0\right)$ gives:

\begin{align}
C = C\_{0}\exp\left(-\sqrt\frac{k}{D}x\right)
\end{align}

where $C\_0$ is the concentration of ammonium in the porewater and thus the concentration at the exterior of the consortium.

Re-arranging to solve for $x$ yields

\begin{align}
x = \frac{-\ln\left(\frac{C}{C\_0}\right)}{\sqrt\frac{k}{D}}
\end{align}

In our approach, we explore $x(C\_0, D)$ holding $C$ constant at 25 µM, the threshold value of ammonium concentration above which diazotrophy is inhibited in incubations of methane seep sediments (Dekas, et al. 2018). $x(C\_0, D)$ will thus describe the depth within a consortium at which ammonium will be depleted sufficiently to induce diazotrophy, described in the figure below as 'diazotrophy frontier depth'. We have calculated $k$ = 0.0004-0.0009 hr$^{-1}$ from time-series measurements of ammonium concentration in methane seep sediment incubations under ammonium-replete (≥ 25 µM) conditions (Dekas, et al. 2018, Fig. S5, "Mat-774", "Mat-794").

In [2]:

```
C = 25
k = 0.0004
```

We explore a range of $C\_0$ between 0.01 and 316 µM, representative of porewater ammonium concentrations measured *in situ* (Dekas, et al. 2018).

In [3]:

```
C_0 = np.logspace(-2, 2.5, 1000)
```

We use an estimate for the diffusivity of ammonium derived from the literature (Krom and Berner, 1980) of $D$ = 3.5 \* 10^6 to constrain maximum possible diffusivity, and explore a range of parameter values for $D$ down to $10^1$, representing the limitations on diffusion imposed by diffusion between cells within the consortium. A large range of values for $D$ was employed here to reflect the challenges of measuring this parameter within ANME-SRB consortia.

In [4]:

```
D = np.logspace(1, 6.7, 1000)
```

In [5]:

```
c_0, d = np.meshgrid(C_0, D)
x = np.zeros((1000, 1000))
x = -np.log(25/c_0) * 1/np.sqrt(k/d)
```

In [7]:

```
fig, ax = plt.subplots()

x = ma.masked_where(x <= 0, x)
norm = cm.colors.LogNorm()
cs = ax.contourf(c_0, d, x, 50,
                 norm = norm)
cbar = fig.colorbar(cs)
plt.xlim(25, c_0.max())
plt.xlabel('Porewater [NH$_4$$^+$] (µM)')
ax.set_yscale('log')
ax.set_xscale('log')
ax.set_xticks([30, 50, 100, 300])
ax.get_xaxis().set_major_formatter(matplotlib.ticker.ScalarFormatter())
plt.ylabel('Diffusivity (µm$^2$ h$^{-1}$)')
cbar.set_label('Diazotrophy frontier depth (µm)')
plt.rcParams['figure.figsize'] = [10, 10]
plt.rcParams['font.size'] = 12
plt.show()
```

Plotting the results, we see that the depth at which we observe significant $^{15}N$ incorporation and thus diazotrophic activity in our consortia (1 to 10 µm into consortia, Fig. 8) is possible at diffusivities near that measured for bulk marine sediment (≈$10^6$ µm$^2$ hr$^{-1}$) for porewater ammonium concentrations that approach 25 µm. Thus, the simple model presented here broadly supports our hypothesized mechanism for the observed gradient in diazotrophic activity presented in Figure 8.
